# HOXD3 was negatively regulated by YY1 recruiting HDAC1 to suppress progression of hepatocellular carcinoma cells via ITGA2 pathway

**DOI:** 10.1111/cpr.12835

**Published:** 2020-06-17

**Authors:** Lumin Wang, Yi Gao, Xiaoge Zhao, Chen Guo, Xiaofei Wang, Yang Yang, Cong Han, Lingyu Zhao, Yannan Qin, Liying Liu, Chen Huang, Wenjing Wang

**Affiliations:** ^1^ Department of Cell Biology and Genetics School of Basic Medical Sciences Xi'an Jiaotong University Health Science Center Xi'an China; ^2^ Key Laboratory of Environment and Genes Related to Diseases Xi'an Jiaotong University Health Science Center Xi'an China; ^3^ Institute of Genetics and Developmental Biology School of Basic Medical Sciences Translational Medicine Institute Xi'an Jiaotong University Health Science Center Xi'an China; ^4^ Yan'an Key Laboratory of Chronic Disease Prevention and Research Yan'an China; ^5^ Cardiovascular Research Center Xi'an Jiaotong University Health Science Center Xi'an China; ^6^ Department of Hepatobiliary Surgery The First Affiliated Hospital of Xi'an Jiaotong University Xi'an China

**Keywords:** cell progression, hepatocellular carcinoma, HOXD3, ITGA2, YY1

## Abstract

**Objectives:**

HOXD3 is associated with progression of multiple types of cancer. This study aimed to identify the association of YY1 with HOXD3‐ITGA2 axis in the progression of hepatocellular carcinoma.

**Materials and Methods:**

Bioinformatics assay was used to identify the effect of YY1, HOXD3 and ITGA2 expression in HCC tissues. The function of YY1 and HOXD3 in HCCs was determined by qRT‐PCR, MTT, apoptosis, Western blotting, colony formation, immunohistochemistry, and wound‐healing and transwell invasion assays. The relationship between YY1 and HOXD3 or HOXD3 and ITGA2 was explored by RNA‐Seq, ChIP‐PCR, dual luciferase reports and Pearson's assays. The interactions between YY1 and HDAC1 were determined by immunofluorescence microscopy and Co‐IP.

**Results:**

Herein, we showed that the expression of YY1, HOXD3 and ITGA2 associated with the histologic and pathologic stages of HCC. Moreover, YY1, recruiting HDAC1, can directly target HOXD3 to regulate progression of HCCs. The relationship between YY1 and HOXD3 was unknown until uncovered by our present investigation. Furthermore, HOXD3 bound to promoter region of ITGA2 and up‐regulated the expression, thus activating the ERK1/2 signalling and inducing HCCs proliferation, metastasis and migration in the vitro and vivo.

**Conclusions:**

Therefore, HOXD3, a target of YY1, facilitates HCC progression via activation of the ERK1/2 signalling by promoting ITGA2. This finding provides a new whole way to HCC therapy by serving YY1‐HOXD3‐ITGA2 regulatory axis as a potential therapeutic target for HCC therapy.

## 1 INTRODUCTION

Hepatocellular carcinoma is considered as the third leading cause of cancer‐related death around the world.^1^ Although the incidence of HCC has reduced notably because of the rapid development of chemotherapy and radiotherapy in the past several decades, the short‐term survival rate is still low and the 5‐year survival is in the range 5%‐30% throughout 2000‐2014 in the world.^2^ Among them, the uncontrolled tumour progression, metastasis and acquired drug resistance are the main reasons. Therefore, it is a pressing requirement to identify the potential new biomarkers for the carcinogenesis and progression of HCC, which is vital for the development of novel therapeutic strategies.

HOXD3 gene belongs to a large homeobox (HOX) superfamily, whose members participate in the regulation of axial regional specification during embryonic development.^3^ Deregulation of HOXD3 had a great effect on tumorigenesis and progression via proliferation, apoptosis, invasion, metastasis and angiogenesis. In breast cancer, HOXD3 highly expressed and went through the integrin β3 to induce breast cancer cell stemness and drug resistance.^4^ Our previous research confirmed that miR‐203a could target HOXD3 3′ UTR region via EGFR and VEGFR to suppress cell progression in SMMC‐7721 and Hep3B cells,^5^ suggesting that HOXD3 was important for tumour development. However, the function of HOXD3 in cancer onset and upstream/downstream regulation maintenance requires further investigation; especially, the relationship between HOXD3 and YY1 in HCCs has rarely been studied.

Yin Yang 1 (YY1) is a member of the GLI‐Kruppel class of zinc finger DNA‐binding proteins and ubiquitously distributed in eukaryotic cells,^6^ which can act as transcriptional activators in several types of cancers, such as lung adenocarcinoma (LUAD), breast cancer, colon and prostate cancer. YY1‐regulated LINC00152 induces triple‐negative breast cancer (TNBC) progression by regulating the stability of PTEN.^7^ As for lung adenocarcinoma, YY1 was significantly higher expressed in LUAD tissues than in non‐tumour tissues at mRNA and protein levels.^8^ YY1 could also promote epithelial‐mesenchymal transition in prostate cancer.^9^ However, YY1 has been proved to suppressed cell proliferation, migration and invasion in pancreatic ductal adenocarcinoma,^10^, ^11^ indicating that YY1 plays the function as the tumour repressors in gene expression regulation. Similarly, YY1, combined with HLJ1, acted as a tumour suppressor in HCC,^12^ whereas YY1 could promote HCC tumorigenicity and inhibit apoptosis of HCC cells through NF‐κB activation.^13^ Hence, the precise role of YY1 in hepatocellular carcinoma progression remains unknown.

Integrins are a group of heterodimeric transmembrane receptors consisted of α and β subunits. Integrin α2 (ITGA2) as a number of α subunit family combines with beta 1 subunit of the α2β1 integrin to form the heterodimer. Accumulating studies demonstrated that the ITGA2 was connected with the proliferation, invasion and metastasis of various cancers, such as gastric cancer^14^ and glioblastoma.^15^ In gastric cancer, inhibition of ITGA2 induced apoptosis and decreased cell migration. Besides, expression of ITGA2 increased tumour cell aggression by up‐regulating PD‐L1 via STAT3 signalling pathway.^16^ From above research findings, the regulation of ITGA2 expression is involved in many genes and cell signalling pathways. However, the underlying molecular mechanisms of the regulation of ITGA2 expression have been elusive in HCC. Meanwhile, the deep understanding of ITGA2 targeted by HOXD3 remained a challenge.

Herein, we revealed that YY1, recruiting HDAC1, directly regulated the progression of Huh7 and HMCC‐97H cells via HOXD3‐related ITGA2/ERK1/2 cell signalling pathways. Increasing or inhibiting the expression of YY1 and HOXD3 demonstrated that YY1 negatively regulated HOXD3 to inhibit proliferation, invasion and migration, and induce apoptosis of HCC cells through directly modulating ITGA2 in vivo and vitro. This is the first report that the relationship between YY1 and HOXD3, HOXD3 and ITGA2 in the regulation of HCC progression. Thus, the YY1‐HOXD3‐ITGA2 interaction can act as a potential therapeutic target in antitumour progression against HCC.

## 2 MATERIALS AND METHODS

### 2.1 Cell culture and HCC tissues

Huh7 and MHCC‐97H cells were purchased from the company (Shanghai Zhong Qiao Xin Zhou Biotechnology Co., Ltd.) and were cultured in DMEM supplemented with 10% foetal bovine serum (FBS) at 37°C and in 5% CO2. Eighty pairs of HCC tissues and counterparts' tissues were collected from the First Affiliated Hospital of Xi'an Jiaotong University, to have enough sample size with statistical significance. No local or systemic treatment had been conducted before the operation. Both tumour and non‐tumour tissues were histologically confirmed. Informed consent was obtained from each patient, and the study was approved by the Institute Research Ethics Committee of The Cancer Center of Xi'an Jiaotong University.

### 2.2 Plasmid and siRNA

siRNAs (YY1 and HOXD3) were chemically synthesized (GenePharma). The plasmid of YY1 and HOXD3 was purchased from the company (Genechem). All the sequences are given in Table S1.

### 2.3 Real‐time PCR

Total RNA was isolated from the tissues and cells using TRIzol reagent (Invitrogen) according to the manufacturer's instructions. qRT‐PCR was performed according to the methods described previously.^17^ All primers used in the present study are shown in Table S1.

### 2.4 RNA‐Seq assay

Total RNA was isolated from the Huh7 cells transfected with HOXD3‐ctrl and HOXD3 using TRIzol reagent (Invitrogen) according to the manufacturer's instructions. The RNA was analysed by KangChen Bio‐tech company.

### 2.5 Cell proliferation and apoptosis assay

Cell proliferation was analysed using the MTT assay. MTT assay was performed according to the methods described previously.^18^

Cell apoptosis was identified using flow cytometry. Forty‐eight hours after transfection, cells were stained with the PI/ FITC‐Annexin V Apoptosis Detection Kit (Abnova), according to the manufacturer's instructions. Apoptotic populations were detected by ModFit software.

### 2.6 Colony formation assay

In brief, transfected Huh7/MHCC‐97H cells were seeded onto 6‐well plates (1000 cells/well). Two weeks later, the plates were stained with 0.1% crystal violet. Then, the plates were washed with PBS three times. Pictures were obtained by ImageJ software.

### 2.7 Wound‐healing assay

HMCC‐97H and Huh7 cells were seeded into 6‐well plates at 85% confluence. A 200‐μL disposable pipette tip was performed to create scratches. Subsequently, the detached cells were removed with PBS, 1% FBS fresh medium was added in the wells, and then the transfection was conducted. Photographs were measured 48 hours after wounding.

### 2.8 Transwell invasion assay

Invasion assays were exhibited by using Transwell chambers. The chambers were coated with Matrigel (BD Biosciences) and maintained in an incubator for 30 minutes. In brief, 2.0 × 10^4^ cells in serum‐free medium were seeded into the upper chamber, and the lower chamber was filled with 600 mL medium supplemented with 10% FBS. After 48 hours, cells that did not migrate via the pores were removed. Cells entered the lower surface of the membrane were fixed with 1% crystal violet. Each test was performed in triplicate.

### 2.9 Western blotting analysis

Western blotting was performed as previously described in detail.^5^ Proteins were extracted from tissues and cells with RIPA lysis buffer containing protease inhibitor (Invitrogen). The protein concentration was calculated with a quantitative analyzer (GeneQuant pro RNA/DNA). Proteins were separated via 8%‐12.5% SDS‐polyacrylamide gel and then transferred to a nitrocellulose membrane, which was incubated with YY1, HOXD3, ITGA2, p‐MEK, p‐ERK, Bcl‐xL, Bad, E‐Cadherin, N‐Cadherin and GAPDH antibody (Cell Signaling Technology; diluted 1/1000). The membranes were washed with TBST and incubated with a goat anti‐mouse/rabbit antibody (Cell Signaling Technology, diluted 1/3000).

### 2.10 Chromatin immunoprecipitation assay (ChIP)

The binding of YY1 to the promoter of HOXD3 and HOXD3 to the promoter of ITGA2 was verified using ChIP analysis. CHIP was performed according to the methods described previously.^19^

### 2.11 Luciferase activity assays

Huh7 cells were seeded in 96‐well plates. After 24 hours, a pGL3 luciferase vector containing the wild‐type or mutated HOXD3/ITGA2 promoter region was co‐transfected with the indicated plasmids, respectively. Luciferase activity assay was conducted using Dual Luciferase Assay System (Promega). Renilla luciferase activity was used as an internal standard.

### 2.12 Immunohistochemistry

Immunohistochemistry was used to identify expression of YY1, HOXD3 and ITGA2 in HCC and counterparts' tissues. Immunohistochemistry was performed as previously described.^20^

### 2.13 Immunofluorescence microscopy

After 48 hours, the transfected HCCs were fixed in 4% formaldehyde, the cells were permeabilized with 0.1% Triton X‐100 and stained using rabbit anti‐YY1(Cell Signaling Technology; diluted 1/500) and mouse anti‐HDAC1 antibody (Cell Signaling Technology; diluted 1/1500), and the cells were measured by a Nikon Eclipse TS100 microscope (Nikon).

### 2.14 Immunoprecipitation

Total proteins were extracted from cells, and 1‐2 μg YY1 antibody was added into 300 μL cell lysates for 30 minutes at 4°C. Then, proteins were incubated with the beads at 4°C overnight. The beads were washed three times with PBS, and then, the samples were analysed by Western blotting.

### 2.15 Tumorigenicity assay in vivo

Briefly, 5‐week‐old male BALB/c‐nude mice were purchased from animal centre of Xi'an Jiaotong University and maintained in accordance with the guidelines of the Animal Care and Use Committee of Xi'an Jiaotong University. Five mice/group were subjected to the experiment to have statistical importance. Tumorigenicity assay was performed according to the methods described previously.^21^

### 2.16 Statistical analysis

Results are shown as mean ± SEM of at least three different experiments. All of the data were analysed using SPSS v.17.0 software (Abbott Laboratories). Difference analysis was assayed by Student's *t* test. The method of ANOVA followed by Tukey's was used to compare the multiple groups. The correlation coefficient (Pearson's) was used to investigate the correlation between YY1 with HOXD3 or ITGA2. *P* values <.05 were considered significant. The YY1, HOXD3 and ITGA2 gene expression in HCC tissues and the data of DFI (disease‐free interval event), PFI (progression‐free interval event), DSS (disease‐specific survival event) and OS (overall survival) were analysed by bioinformatics. All methods and websites are given in Table S2.

## 3 RESULTS

### 3.1 YY1 inhibits progression and migration of HCCs in vitro

**FIGURE 1:**
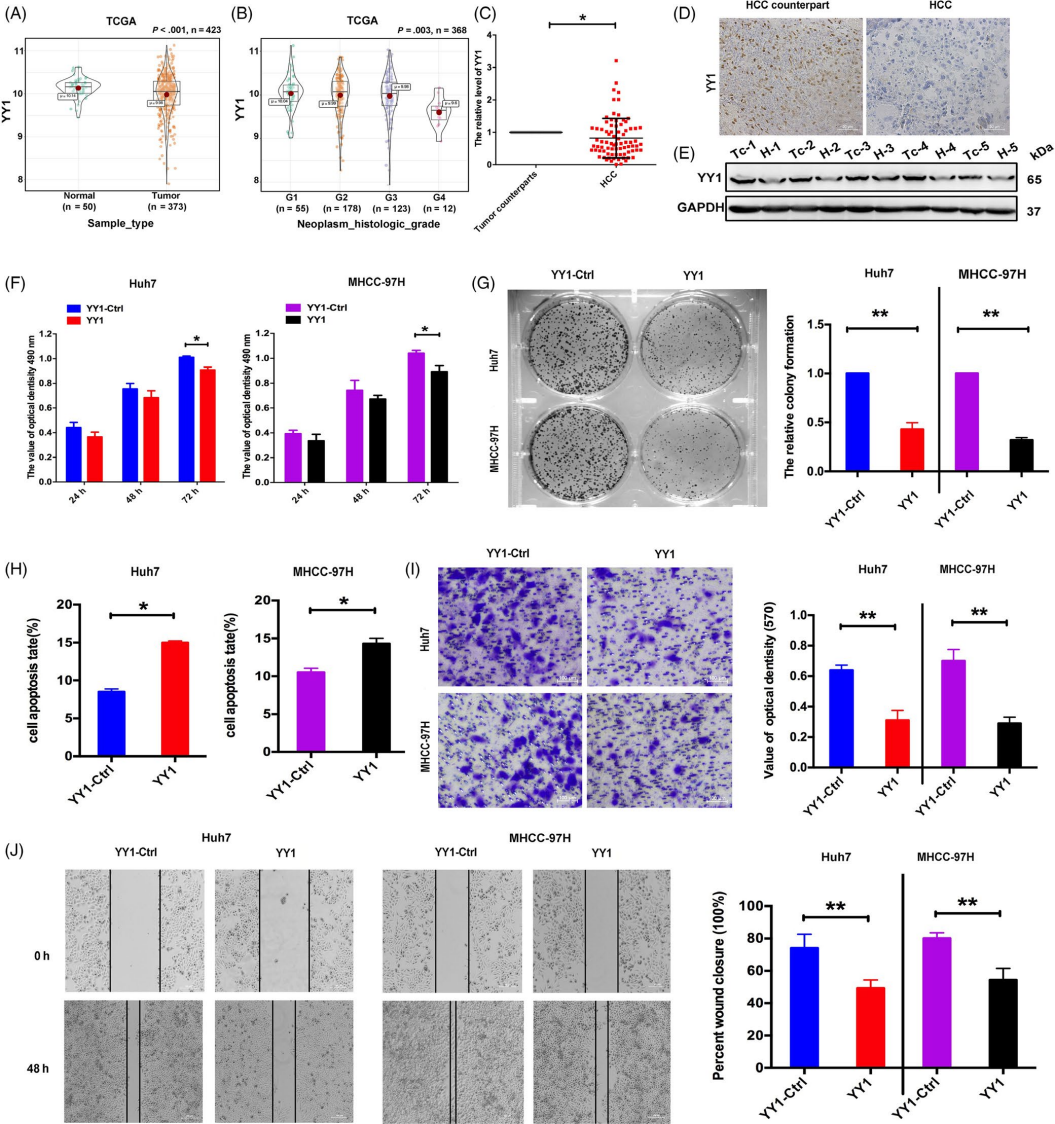
YY1 inhibited the progression and induced apoptosis of HCCs. A, TCGA database showed YY1 expression in HCC tissues and their normal tissues. B, The expression of YY1 connected with clinicopathologic characteristics of HCC patients. C, YY1 mRNA expression in HCC tissues vs counterparts' tissues. D, E, YY1 expression was identified in HCC tissues and their counterparts' tissues at the protein level by using Western blotting and immunohistochemistry assays. F, MTT assay was performed to determine the growth of HCCs treated with YY1 overexpression construct or negative control. G, The colony formation assay was performed several days after the transfection of HCCs with YY1 or negative control. H, Apoptosis was determined in HCCs at 48 h after transfection with YY1. I, Transwell analysis of HCCs after transfected with YY1‐Ctrl and YY1. Quantitative analysis of the invasion rates by solubilization of crystal violet and spectrophotometric reading at OD 570 nm. J, Wound‐healing assays with HCCs treated with YY1‐Ctrl and YY1. The relative wound closure (100%) represents the metastasis capacity of HCCs (the results are expressed as mean ± SEM; *P < .05, **P < .01)

TCGA database was performed to identify the effect of YY1 expression in HCC tissues. Figure 1A,B and Table 1 show that YY1 was lower expressed in HCC tissues than in normal tissues (*P* < .001) and the expression connected with neoplasm histologic grade of HCC patient specimens (*P* = .003). Low levels of YY1 expression were correlated with poor tumour histology (well: 44.8%; moderate: 71.9%; poor: 84.2%). Furthermore, we identified the expression of YY1 in 80 paired HCC tissues and normal tissues by qRT‐PCR. Compared with normal tissues, YY1 was found to be significantly down‐regulated in 65% (52/80) of HCC samples (Figure 1C). Meanwhile, the expression patterns of the YY1 at the protein level in HCC tissues were also examined by IHC. YY1 was lower expressed in HCC tissues than in normal tissues (Figure 1D). Compared with 5 pairs of HCC normal counterparts, the expression of YY1 was down‐regulated in their HCC tissues (Figure 1E).

**TABLE 1:** Patient characteristics and clinicopathologic correlation of YY1, HOXD3 and ITGA2 expression

Characteristics	Number of cases	YY1 expression		*P*‐value	ITGA2 expression		*P*‐value	HOXD3 expression		*P*‐value
		High	Low		High	Low		High	Low	
Age (y)										
≥60	42	13	29	.49	33	9	.61	30	12	.43
＜60	38	15	23		28	10		24	14	
Gender										
Male	44	19	25	.10	31	13	.18	26	18	.08
Female	36	9	27		30	6		28	8	
pTNM stage										
I	22	9	13	.89	19	3	.35	13	9	.14
II	30	10	20		21	9		25	5	
III	17	5	12		14	3		10	7	
IV	11	4	7		7	4		6	5	
Histology										
Well	29	16	13	.01	16	13	.01	14	15	.02
Moderate	32	9	23		28	4		26	6	
Poor	19	3	16		17	2		14	5	

To investigate the role of YY1 in HCCs, gain and loss of function of YY1 assays were used. The expression of YY1 was markedly increased in the YY1 transfected MHCC‐97H and Huh7 cells compared to the control vector transfected cells (Figure S1A,B). MTT was applied to identify the role of YY1 in HCC progression. Results revealed that overexpression of YY1 decreased the proliferation of HMCC‐97H and Huh7 cells at 72 hours (Figure 1F). Furthermore, a colony formation assay was used to investigate the inhibitory role of YY1 in HMCC‐97H and Huh7 cells. Compared with control transfected cells, fewer and smaller colonies had been found in YY1 transfected cells significantly (Figure 1G). Additionally, YY1 promoted apoptosis of HCCs in YY1 transfected HMCC‐97H and Huh7 cells, compared to cells transfected negative control vector (Figure 1H). Meanwhile, wound‐healing and transwell migration and invasion assays were used to verify the function of YY1 on HCC migration and invasion (Figure 1I,J). The results show that YY1 decreased the migration and invasion of HCCs.

**FIGURE 2:**
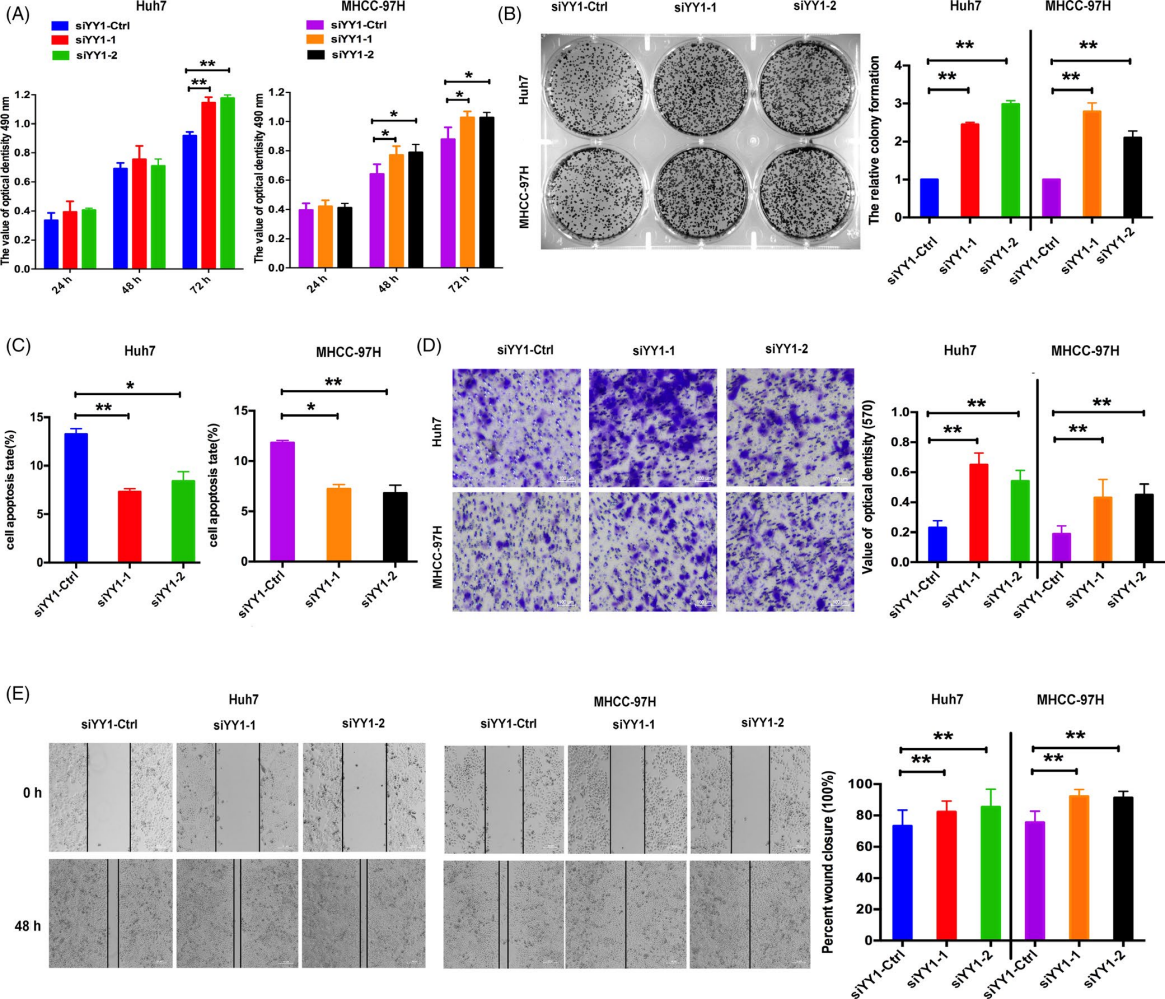
Inhibitor of YY1 increased progression and reduced apoptosis of HCCs. A, MTT assay of Huh7/MHCC‐97H cells after inhibiting the expression of YY1. B, The growth of Huh7/MHCC‐97H cells was detected by colony formation. The colony formation assay was determined in HCCs transfected with siYY‐1, siYY1‐2 or negative control (siYY1‐Ctrl). C, Apoptosis was determined in HCCs transfected with siYY‐1, siYY1‐2 or a negative control. D, E, Transwell and wound‐healing analysis represented the migration and metastasis capacity of HCCs (the results are expressed as mean ± SEM; *P < .05, **P < .01)

To further confirming that YY1 played the role of tumour inhibitor, the YY1 siRNA or its control was transfected into Huh7 and HMCC‐97H. Using the MTT, colony formation and cell apoptosis, inhibition of YY1 expression increased HCCs progression and decreased the apoptosis of HCCs (Figure 2A‐C). Inhibition of YY1 expression induced the migration and invasion of HCCs (Figure 2D,E), which strongly suggested that YY1 acted as a tumour suppressor in HCCs.

### 3.2 HOXD3 induces proliferation and metastasis and inhibits apoptosis of HCCs in vitro

**FIGURE 3:**
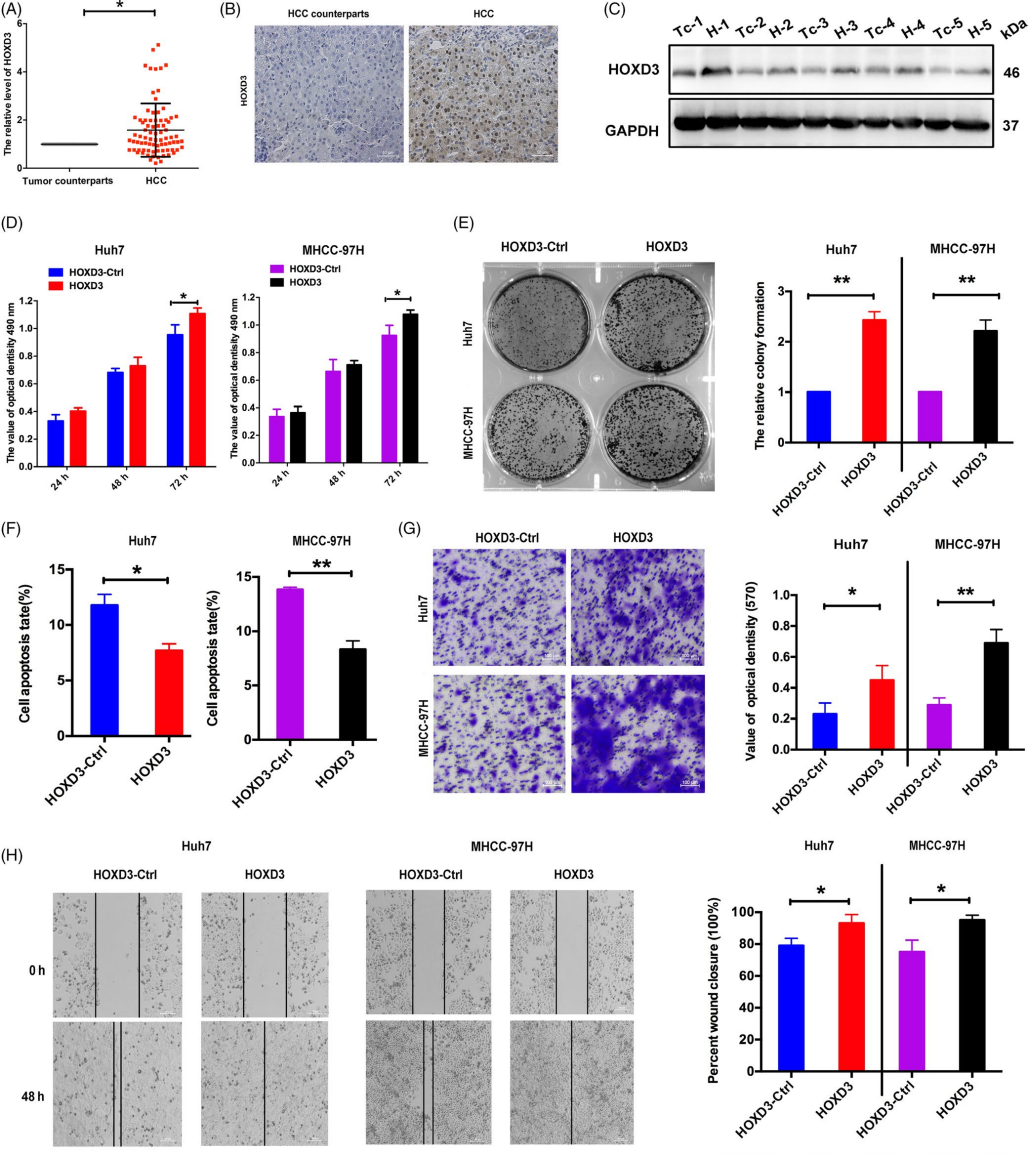
HOXD3 induced the progression and induced apoptosis of HCCs A, The expression of HOXD3 in HCC tissues and their counterparts at RNA level. B, C, HOXD3 protein expression in HCC tissues vs counterparts' tissues was confirmed by using Western blotting and immunohistochemistry assays. D, The effects of HOXD3 on HCC proliferation were determined by MTT assay at 24, 48 and 72 h. E, Representative results of colony formation of HCCs after HOXD3 overexpression. F, Cell apoptosis determined in HCCs 48 h after transfection. G, Transwell analysis of HCCs after transfected with HOXD3‐Ctrl and HOXD3. Quantitative analysis of the invasion rates by solubilization of crystal violet and spectrophotometric reading at OD 570 nm. H, Wound‐healing assays with HCCs treated with HOXD3‐Ctrl and HOXD3. The relative wound closure (100%) represents the metastasis capacity of HCCs (the results are expressed as mean ± SEM; *P < .05, **P < .01)

TCGA database was used to elucidate the effect of HOXD3 in HCC tissues, Figure S2A‐C reveals that the expression of HOXD3 was higher in HCC tissues than counterparts and the expression associated with the metastasis status and cancer stages of HCC. Furthermore, higher expression of HOXD3 induced the lower survival rate (Figure S3A‐D). The expression of HOXD3 was related with the DFI (disease‐free interval event, *P* = .0018), PFI (progression‐free interval event, *P* = .0023), DSS (disease‐specific survival event, *P* = .011) and OS (overall survival, *P* = .038) of HCC. It is worth noting that HOXD3 expression levels were extremely connected with tumour histology (well: 48.2%; moderate: 81.3%; poor: 73.7%), but not associated with age and gender (Table 1). Using the qRT‐PCR assay, HOXD3 was higher expressed in HCC tissues than in normal tissues (Figure 3A). Western blotting and IHC assays also indicated that the HOXD3 was obviously up‐regulated in HCC tissues than in their counterparts at the protein level (Figure 3B,C). Together, these results showed that high HOXD3 expression associates with higher HCC stages.

**FIGURE 4:**
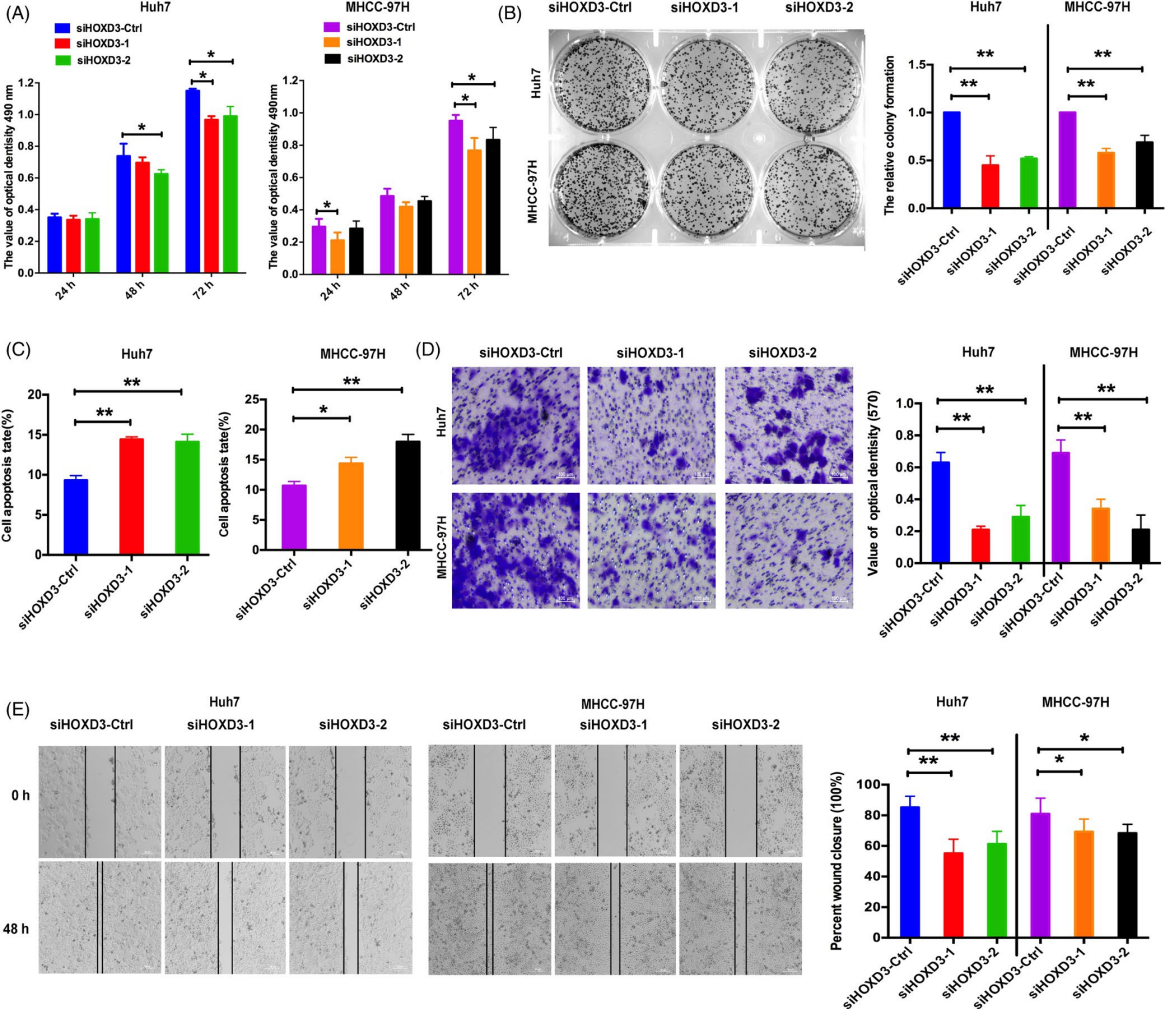
Inhibition of HOXD3 decreased the HCC proliferation, migration and increased apoptosis. A‐C, MTT, colony formation and cell apoptosis assays were performed to determine the impact of HCCs treated with siHOXD3‐1, siHOXD3‐2 or negative control. D, E, Transwell and wound‐healing analysis represented the migration and metastasis capacity of HCCs transfected with siHOXD3‐Ctrl, siHOXD3‐1 and siHOXD3‐2 (the results are expressed as mean ± SEM; *P < .05, **P < .01)

To investigate the role and function of HOXD3 in Huh7 and MHCC‐97H cells, HOXD3 expression level has been identified by qRT‐PCR. HOXD3 expression was improved in HCCs transfected with HOXD3 expression vector, and the HOXD3 expression inhibited by two RNA interference (small interfering RNA [siRNA]) in HCCs (Figure S4A,B). MTT, colony formation, cell apoptosis, and wound‐healing and transwell assays were applied to identify the role of HOXD3 in the progression, invasion and migration of HCCs (Figure 3D‐H). Contrary to the effect of YY1 in HCCs, HOXD3 induced cell progression, migration and invasion, and inhibited the apoptosis. Silencing of HOXD3 led to inhibition of cell proliferation, invasion, migration, and the promotion of cell apoptosis, which was in line with the effects of YY1 in HCCs (Figure 4A‐E). These results demonstrated that HOXD3 played the key role of an oncogene in HCCs progression.

### 3.3 HOXD3 is directly targeted by YY1

**FIGURE 5:**
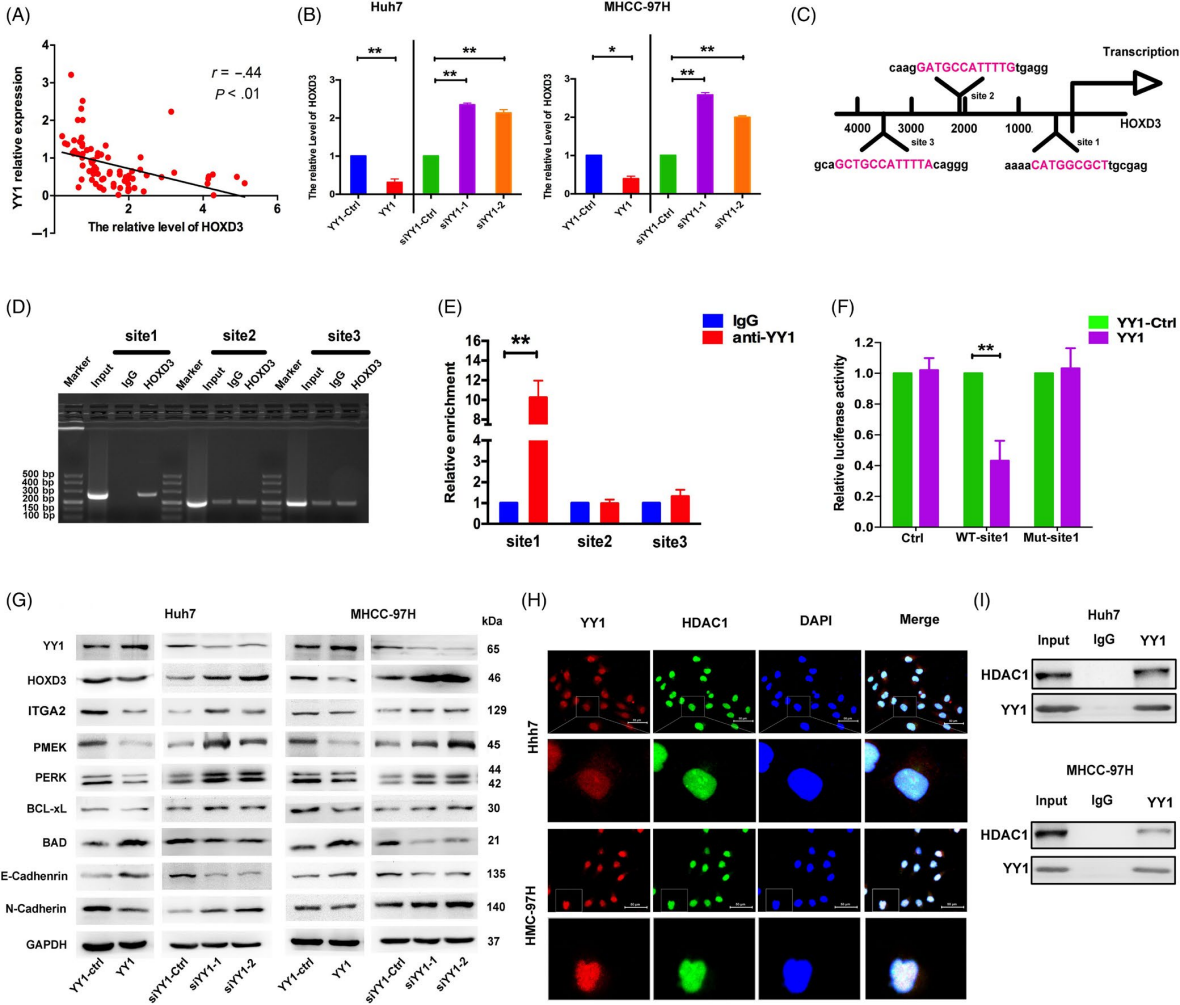
YY1 activates HOXD3 expression directly. A, The relationship between YY1 and HOXD3 was assayed by Pearson's *r*. B, The expression levels of HOXD3 were determined by qRT‐PCR in HCCs transfected with YY1 or siYY1. C, Schematic diagram of the putative HOXD3 promoter with one potential YY1 response element. D, The interaction of YY1 with HOXD3 was shown using ChIP assays with control (rat IgG) or anti‐YY1 antibody. E, qRT‐PCR analysis was performed with primers spanning predicted YY1 of HOXD3. F, Luciferase assays were performed in Huh7 cells transfected with wt or mut promoter. G, The expression of HOXD3, ITGA2, p‐MEK, p‐ERK, Bcl‐xL, Bad, E‐cadherin and N‐cadherin was detected by Western blotting. H, The expression of YY1 and HDAC1 was detected by immunofluorescence staining. I, The expression of YY1 and HDAC1 was detected using Co‐IP (the results are expressed as mean ± SEM; *P < .05, **P < .01)

The qRT‐PCR result confirmed the levels of YY1 were negatively associated with HOXD3 expression in HCC tissues (Figure 5A). Likewise, YY1 inhibited the expression of HOXD3 in the Huh7 and MHCC‐97H cells at the RNA level (Figure 5B). Meanwhile, the UCSC genome browser tool and JASPAR website were used to predict the binding sequences of YY1 on HOXD3. There were putative binding sequences for YY1 located at 0.3, 2.4 and 3.7 kb upstream of the HOXD3 locus, respectively (Figure 5C). Based on the bioinformatics result, the CHIP‐PCR analysis was used to study the effect of YY1 in the biological function of HCCs by directly targeting HOXD3, the result indicated that YY1 target the promoter region of HOXD3 at 0.3 kb (Figure 5D,E). Luciferase reporter analysis showed that overexpression of YY1 led to an decrease in luciferase activity of the wt‐HOXD3‐promoter plasmid in Huh7 cells, while mut YY1 binding site attenuated the decrease of luciferase activity (Figure 5F).The Western blotting assay also indicated that the overexpression of YY1 inhibited the HOXD3, ITGA2, p‐MEK, p‐ERK, Bcl‐xL, N‐cadherin expression and increased Bad, E‐cadherin (Figure 5G and Figure S5). In order to confirm the negative correlation between YY1 and HOXD3, co‐localization of YY1 and HDAC1 expression was measured by immunofluorescence staining and coimmunoprecipitation (Co‐IP) assays (Figure 5H,I), suggesting that YY1, combined with HDAC1, contributed to proliferation, migration and invasion inhibition, apoptosis promotion by directly modulating HOXD3 in the HCCs.

### 3.4 Overexpression of HOXD3 eliminates the effects of YY1 on HCCs

**FIGURE 6:**
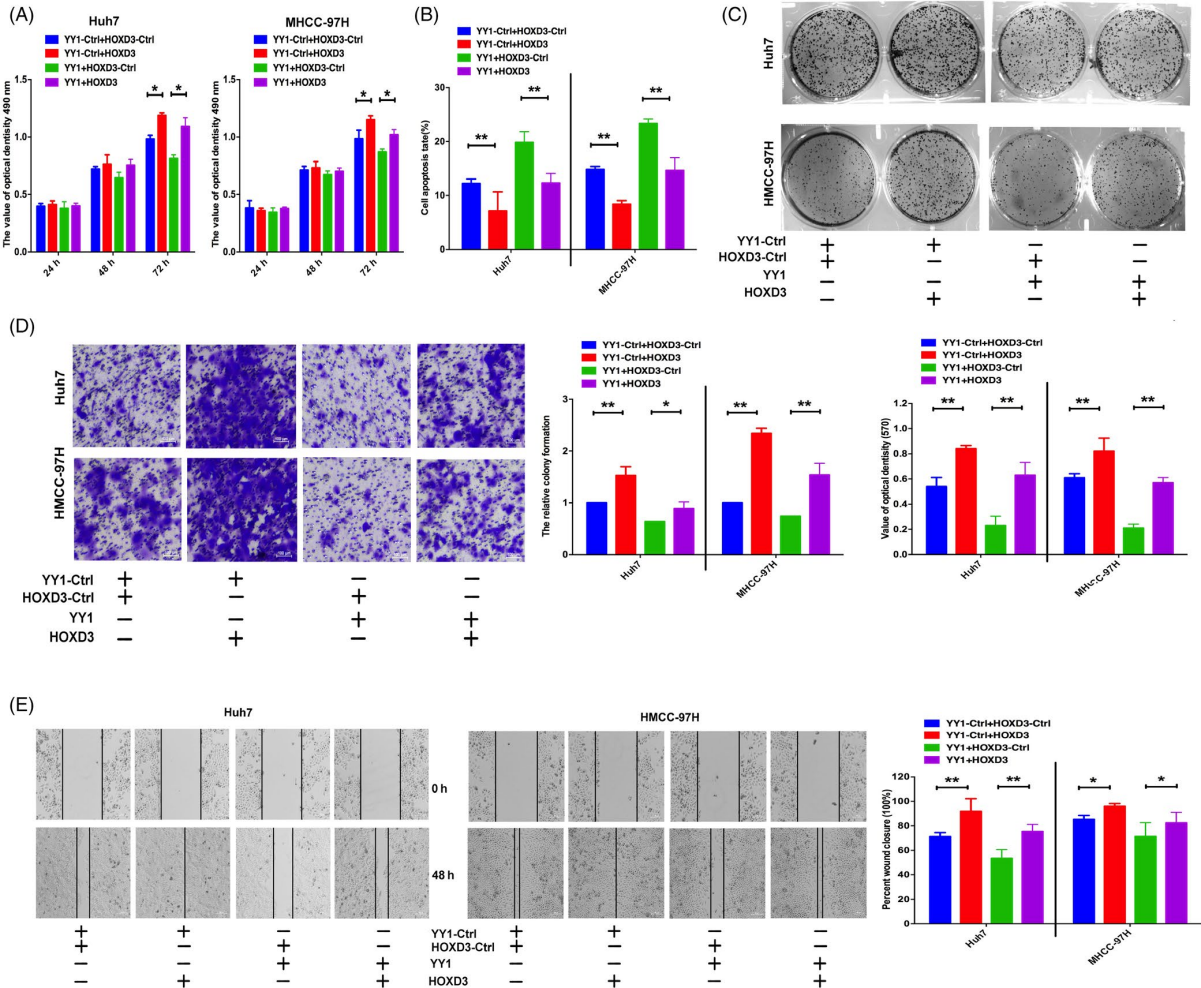
YY1 rescues HOXD3 induced cellular invasion in HCCs. A‐C, MTT, colony formation and cell apoptosis assays were performed to determine the impact of HCCs treated with HOXD3 and YY1 or negative control. D, E, Transwell and wound‐healing analysis represented the migration and metastasis capacity of HCCs co‐transfected with HOXD3 and YY1‐Ctrl or YY1 (the results are expressed as mean ± SEM; *P < .05, **P < .01)

To further confirm that the YY1 played the role of tumour suppressor through HOXD3, HOXD3 was co‐transfected with YY1‐Ctrl or YY1 into HCCs. HOXD3 reversed the tumour proliferation–suppressing effect of YY1 on HMCC‐97H and Huh7 cell proliferation (Figure 6A). Meanwhile, overexpression of HOXD3 induced the colony formation and decreased cell apoptosis in Huh7/HMCC‐97H cells (Figure 6B,C). Additionally, increased expression of HOXD3 induced migration and invasion of Huh7 and HMCC‐97H cells. However, the expression of YY1 reduced these effects of HOXD3 on HCCs after co‐transfection of YY1 and HOXD3 (Figure 6D,E). These results further suggest that YY1 plays tumour suppressor role by directly targeting at HOXD3.

### 3.5 HOXD3 directly targets ITGA2 to affect the progression of HCCs in vitro

**FIGURE 7:**
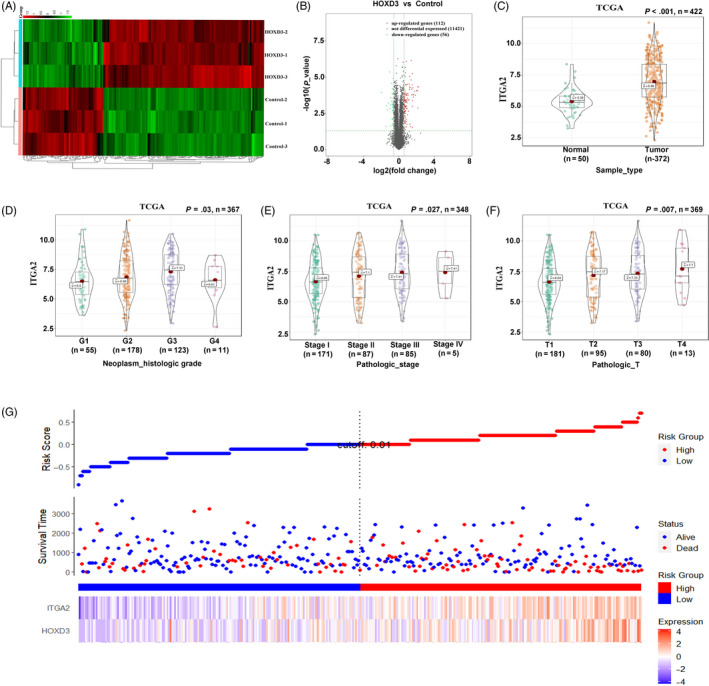
ITGA2 higher expressed in HCC tissues and connected with tumour migration. A, Heat map diagram of differential mRNA expression profiles between HOXD3‐ctrl and HOXD3. B, Differential mRNA expression profiles between HOXD3‐ctrl and HOXD3. Red = genes with higher expression, green = genes with lower expression and grey = gene with not different expression. C, The expression of ITGA2 in HCC tissues and adjacent noncancerous liver tissues at RNA in TCGA database. D‐F, The expression of ITGA2 connected with pathologic and histologic grade of HCC patients. G, The expression of ITGA2 connected with survival rate of HCC patients

Using the RNA‐Seq assay, 112 genes were up‐regulated when HOXD3 transfected in Huh7 cells and 56 genes were down‐regulated compared with negative control. Among the up‐regulated genes, ITGA2 was higher expressed in Huh7 cells (Figure 7A,B). Furthermore, bioinformatics analysis was applied to identify the function of ITGA2 in HCC tissues. ITGA2 was higher expressed in HCC tumour than in normal tumour (Figure 7C), and the expression associated with the histologic and pathologic stages of HCC (Figure 7D‐F). Meanwhile, high expression of ITGA2 was related with patient survival rate (Figure 7G and Figure S6). The expression of ITGA2 was induced lower DFI (*P* = .0073), PFI (*P* = 0004) and OS (*P* = .011) of HCC.

**FIGURE 8:**
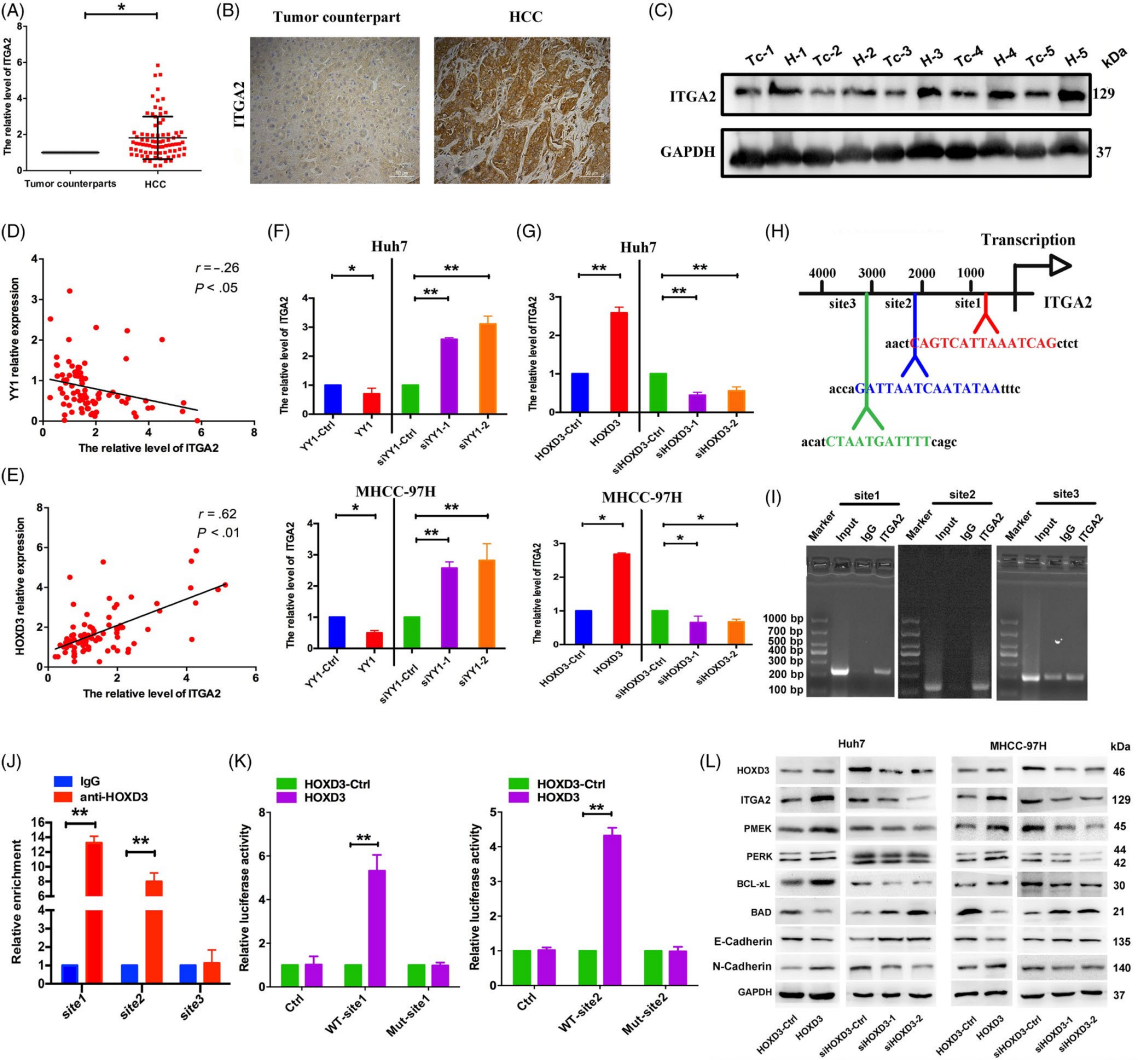
HOXD3 targets the promoter region of ITGA2 directly and regulates the expression of ITGA2. A, The expression of ITGA2 in 80 pairs HCC tissues and adjacent noncancerous liver tissues at RNA level. B, C, ITGA2 protein expression in HCC tissues vs counterparts was identified by using immunohistochemistry and Western blotting assays. D, E, Pearson's *r* was used to test the relationship between HOXD3 and ITGA2, YY1 and ITGA2. F, G, The expression of ITGA2 in HCCs transfected with HOXD3/YY1 or siHOXD3/si‐YY1. H, Schematic diagram of the putative ITGA2 promoter with one potential HOXD3 response element. I, J, The interaction of HOXD3 with ITGA2 was shown using ChIP‐qRT‐PCR assays with control (IgG) or anti‐HOXD3 antibody. K, Luciferase activity relative to Renilla control was measured in Huh7 cells. L, The expression of ITGA2, p‐MEK, p‐ERK, Bcl‐xL, Bad, N‐cadherin and E‐cadherin was detected by Western blotting (the results are expressed as mean ± SEM; *P < .05, **P < .01)

In order to investigate the function of ITGA2, qRT‐PCR, Western blotting and IHC were performed to test the expression of ITGA2 in the HCC tissues at the RNA and protein levels. ITGA2 expression was increased in HCC tissues than normal tissues (Figure 8A‐C). Furthermore, the higher expression level of ITGA2 was related to histology of HCC patients (well: 44.8%; moderate: 87.5%; poor: 89.5%) (Table 1). Pearson's *r* showed that HOXD3 expression was positively correlated with ITGA2 expression and YY1 expression was negatively correlated with ITGA2 expression (Figure 8D,E). Meanwhile, inhibiting the expression of HOXD3 repressed ITGA2 and overexpression of YY1 inhibited the expression of ITGA2 in Huh7 and MHCC‐97H cells (Figure 8F,G). To further investigate the relationship between HOXD3 with ITGA2, the UCSC genome browser tool, VISTA and the JASPAR database were analysed, and a putative HOXD3‐binding site located at 0.7, 2.1 and 3.1 kb, respectively, upstream of the ITGA2 gene in Figure 8H. The ChIP‐PCR experiment showed the same result that HOXD3 bond to the putative binding site 0.7 and 2.1 kb upstream of ITGA2 (Figure 8I,J). In addition, the effect of HOXD3 on ITGA2 transcriptional activity was identified by using double‐luciferase reporter assays. The results showed that ITGA2 activity was significantly highly regulated in HOXD3‐overexpressing cells compared with control cells (Figure 8K). Furthermore, overexpression of HOXD3 increased the expression levels of ITGA2, p‐MEK, p‐ERK, Bcl‐xL and N‐cadherin, but decreased the expression level of Bad and E‐cadherin (Figure 8L and Figure S7). After silencing of HOXD3, the expression levels of ITGA2, p‐MEK, p‐ERK, Bcl‐xL and N‐cadherin were suppressed, while the expression levels of Bad and E‐cadherin were promoted, which suggested that HOXD3 contributed to the progression, apoptosis inhibition by directly modulating ITGA2 transcription in HCCs.

### 3.6 Inhibition of HOXD3 reduces growth and migration of Huh7 cells in vivo

**FIGURE 9:**
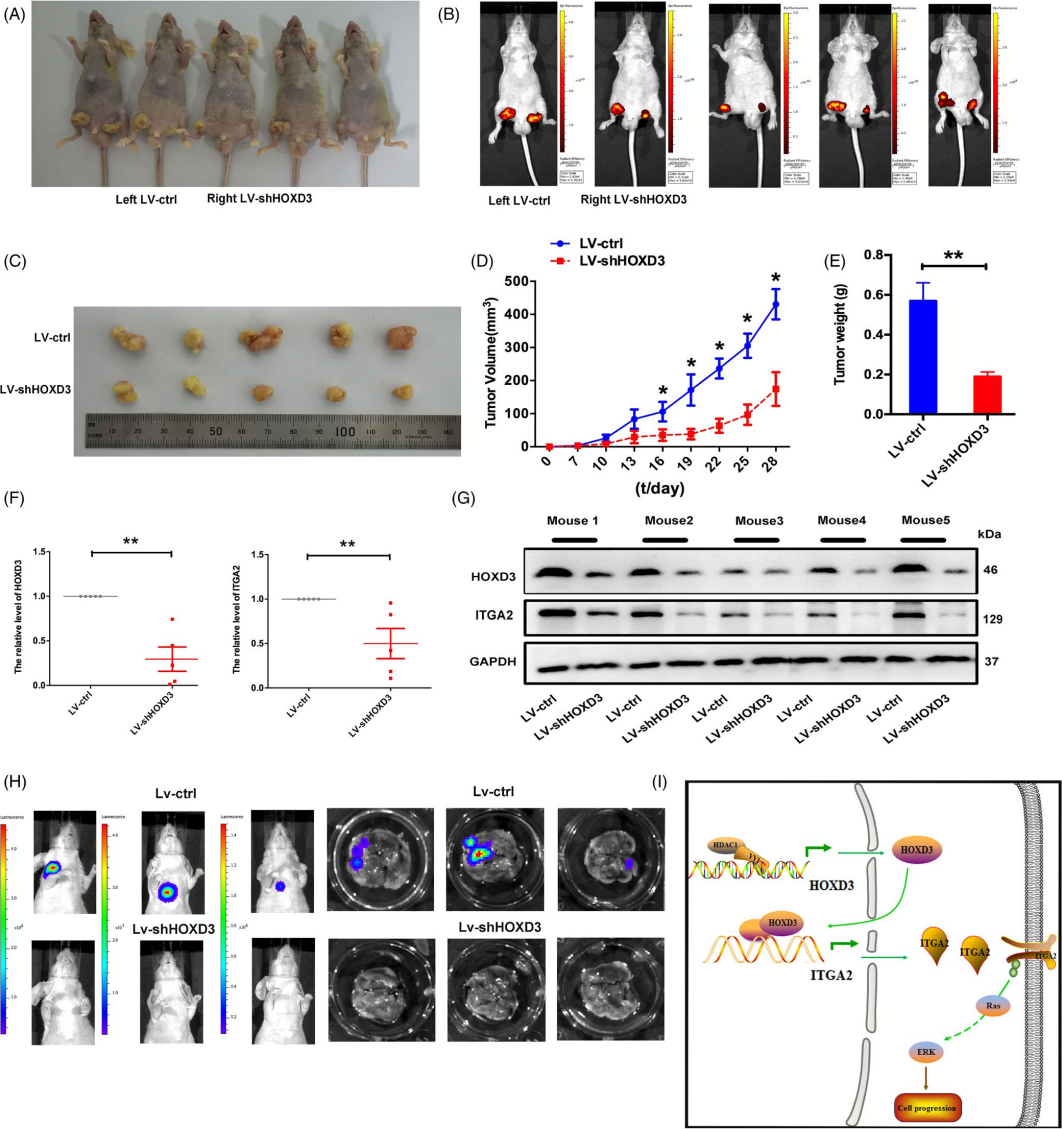
Silencing HOXD3 expression inhibits hepatocellular carcinoma progression in vivo. A, Gross morphology of tumours injected with either LV‐ctrl or LV‐shHOXD3 cells after 28 d. B, Small animal imaging analysis was used to assess tumour volume in situ at day 28 during tumour development. C, Morphology of excised tumours from nude mice. D, Growth curves of tumour volume were generated every 3 d for 28 d. E, Tumour weight. F, G, The expression levels of HOXD3 and ITGA2 were analysed by RT‐PCR and Western blotting in tissues from the animal. H, The metastasis of huh7 cells suppressing HOXD3 was shown. Down‐expression of HOXD3 significantly inhibited the metastasis of huh7 cells to the lung. I, Proposed model for the effects of YY1‐mediated HOXD3 on HCC progression via regulation of the ITGA2–ERK1/2 signalling pathway (the results are expressed as mean ± SEM; *P < .05, **P < .01)

To investigate the function and role of HOXD3 in vivo, subcutaneous injected transplantation tumour researches were exhibited in nude mice. Tumour nodules were measured every 3 days for 4 weeks. The tumour growth was obviously inhibited by LV‐shHOXD3 compared with the LV‐ctrl (Figure 9A‐C). On the last day, the mean volume of tumours injected with LV‐shHOXD3 was much lower than those of control tumours (n = 5, Figure 9D). In addition, that mice injected with HOXD3‐downexpressing cells formed smaller tumours compared with the controls (Figure 9E). Furthermore, the expression of HOXD3 and ITGA2 in LV‐shHOXD3 tumours was lower than that in control tumours (Figure 9F), and the expression of HOXD3 and ITGA2 was notably decreased in the HOXD3‐downexpressing treated tumours as compared with that in the control treated tumours at protein levels (Figure 9G). Meanwhile, intravenous injection experiment was adopted to determine the effect of HOXD3 on the metastasis of HCC cells in vivo. In contrast with the LV‐NC group, all mice in LV‐shHOXD3 group formed lower liver metastasis (Figure 9H). Based on the above experimental result, we concluded that HOXD3 promoted HCC proliferation via increasing ITGA2 in vivo (Figure 9I).

## 4 DISCUSSION

Herein, YY1 had been known to have two sides, a tumour promoter and a tumour suppressor, in various kinds of cancer types, such as breast cancer and lung cancer, depending on the cancer type and its interacting partners by regulating the expression or repression of related genes.^22^ In HCC, the tumour suppressor HLJ1 expression was up‐regulated via YY1,^12^ whereas YY1 may promote tumour progression by regulating QKI.^23^ In our research, we found that YY1 could suppress tumour progression by directly targeted HOXD3 via ITGA2‐ERK cell signalling. An insight into the mechanism by which YY1 functions as a tumour promoter vs tumour repressor would help us design novel therapeutic approaches by targeting or manipulating the function of YY1. Using the bioinformatics analysis, we showed that YY1 was lower expressed in HCC tissues than their counterparts, which was consistent with results of the website of (http://merav.wi.mit.edu/), and this result had been identified in 80 paired HCC tissues in our researches. From gain‐ and loss‐of‐function assays, we further verified that YY1 could target the HOXD3 via ITGA2‐ERK signalling to suppress the progression and migration of HCCs in vitro (Figures 1 and 2). Whereas YY1 was higher expressed in QGY 7701, QGY 7703 and HepG2 cells and played the role of oncogene^24^, ^25^ in HCC, the different research models might contribute to the inconsistency.

In the human genome, 39 HOX genes are arranged into four different chromosomes. HOX is a transcription factor, which is known to be involved in early embryonic development and body segmentation.^26^ In the previous study, HOX genes are found in the development of various cancers, such as head and neck cancer acute myeloid leukaemia,^27^ ovarian cancer^28^ and bladder cancer.^29^ The dysregulated expression of HOXD, as a member of HOX family, has been identified in solid tumours, including testicular germ cell tumours,^30^ ewing sarcoma^31^ and breast cancer.^32^ The research of ewing sarcoma exhibited that HOXD11 and HOXD13 promoted growth and metastasis of ewing sarcoma. Our previous researches demonstrated that HOXD3 induced proliferation and migration of SMMC‐7721 and Hep3B cells via targeting EGFR and VEGFR.^5^, ^33^ As shown in Figures 3 and 4 and Figure S4, HOXD3 was higher expressed in HCC tissues than the counterparts' tissues, and disease‐free interval, progression‐free interval, disease‐specific survival and overall survival were attributed to the high expression of HOXD3, suggesting that HOXD3 plays the role of prognostic biomarkers in HCC. Meanwhile, gain and loss of function of HOXD3 assays demonstrated that HOXD3 inhibited HCCs apoptosis, promoted the proliferation, invasion and metastasis of HCCs, suggesting HOXD3 played the key role of an oncogene in HCC progression.

Meanwhile, using the assay of bioinformatics database and CHIP‐PCR, YY1 directly bound to the HOXD3 promoter region at 0.3 kb (Figure 5). Interestingly, in our present researches, the expression of YY1 was negatively correlated with HOXD3. Previous evidence showed that YY1 could carry out transcriptional repression by complexing with corepressors, such as HDAC1 and HDAC2.^34^, ^35^ Therefore, we hypothesized that the expression of YY1 negatively correlated with that of HOXD3 might be the result of the recruitment of HDACs with YY1. To investigate the regulatory effect of YY1 on HOXD3, immunofluorescence staining and Co‐IP experiments were performed in HCC cells. Endogenous YY1 coimmunoprecipitated with endogenous HDAC1 in HCC cells, suggesting that it was HDAC1 that might be physically associated with YY1 and contributed to the suppressive activity. This is the first report that a relationship between YY1 and HOXD3 has been confirmed in HCC cells, and HOXD3 can be regulated directly by YY1 to suppress proliferation and migration and promote apoptosis. In addition, HOXD3 could reverse the progression effect of YY1 on HCCs (Figure 6). Thus, these results robustly suggest that HOXD3 down‐regulation induced by YY1 can inhibit the progression of HCC cells.

In order to investigate the HOXD3 regulatory mechanism in HCCs, bioinformatics, RT‐PCR, CHIP‐PCR and dual luciferase reports assays were used. HOXD3 levels were associated with ITGA2 expression levels, and that HOXD3 could bind to the promoter region of ITGA2 and regulate HCC progression by directly targeting ITGA2 (Figure 8). It is well known that ITGA2, as an oncogene, participates in biological processes of cancers, such as progression, invasion, metastasis and angiogenesis.^36^, ^37^ As for acute myeloid leukaemia (AML), overexpression of ITGA2 was connected with lower 5‐year survival.^38^ ITGA2 was targeted by miR‐16‐5p, suppressed tumour proliferation, angiogenesis in the colorectal cancer.^39^ Our present research revealed that the ITGA2 was higher expressed in HCC tissues than in normal tissues, and high ITGA2 expression brought about low short‐term survival rates and poor prognosis of HCC patients (Figure 7). Meanwhile, the ITGA2 expression level connected with the patient clinicopathologic in the HCC tissues. Furthermore, ITGA2 affected the biological processes of HCC cells with ERK1/2 involved cell signalling pathway, which was consistent with the research of ITGA in salivary adenoid cystic carcinoma (SACC),^40^ breast cancer,^41^, ^42^ pancreatic cancer^43^ and gastric canceryy.^44^ In the research of gastric cancer, miR‐135b‐5p could target ITGA2 via ERK pathway to regulate the chemoresistance of the gastric cancer cells. Meanwhile, in vivo studies exhibited that silencing the expression of HOXD3 could inhibit the growth of Huh7 cells and suppress the expression of ITGA2 in Figure 9, which greatly supported our observations that HOXD3 directly targeted ITGA2 and induced HCC growth in vitro.

In conclusion, the present study demonstrates that YY1 acts as a tumour suppressor in the progression of HCC with a study of YY1 gain and loss of function. YY1 combined with HDAC1 to target promoter region of HOXD3 and inhibit the expression of HOXD3. Furthermore, the molecular mechanism was performed via exhibiting an merged approach with an association of bioinformatics, RNA‐Seq, Pearson correlation coefficient and chromatin immunoprecipitation PCR assay, which suggested that HOXD3 played the role of prognostic biomarkers and promoted proliferation, migration and metastasis and inhibited apoptosis of HCCs by directly targeting ITGA2 through ERK1/2 involved cell signalling pathway. Our findings indicate that YY1‐HOXD3‐ITGA2 interaction plays a critical role in HCC progression and can act as a potential novel therapeutic target for HCC therapy.

## CONFLICT OF INTEREST

The authors declare no conflict of interest.

## AUTHOR CONTRIBUTIONS

Lumin Wang and Yi Gao contributed equally to this work. Lumin Wang and Chen Huang conceived and designed the study. Yang Yang, Wenjing Wang, Yannan Qin, Chen Guo and Xiaoge Zhao acquired the data. Yi Gao, Cong Han, lingyu Zhao and Liying Liu analysed and interpreted the data. Lumin Wang drafted the manuscript. Chen Huang critically revised the manuscript for important intellectual content. Xiaofei Wang provided administrative, technical and material support. All authors read and approved the final manuscript.

## Supporting information

Fig S1

Fig S2

Fig S3

Fig S4

Fig S5

Fig S6

Fig S7

Table S1

Table S2

Supplementary Material

## Data Availability

The data that support the findings of this study are available from the corresponding author upon reasonable request.
