# Supplementary figures and images for: HOXD3 was negatively regulated by YY1 recruiting HDAC1 to suppress progression of hepatocellular carcinoma cells via ITGA2 pathway
Source: Cell Prolif. 2020 Jun 17;53(8):e12835. doi: 10.1111/cpr.12835 (PMC7445403; doi:10.1111/cpr.12835)

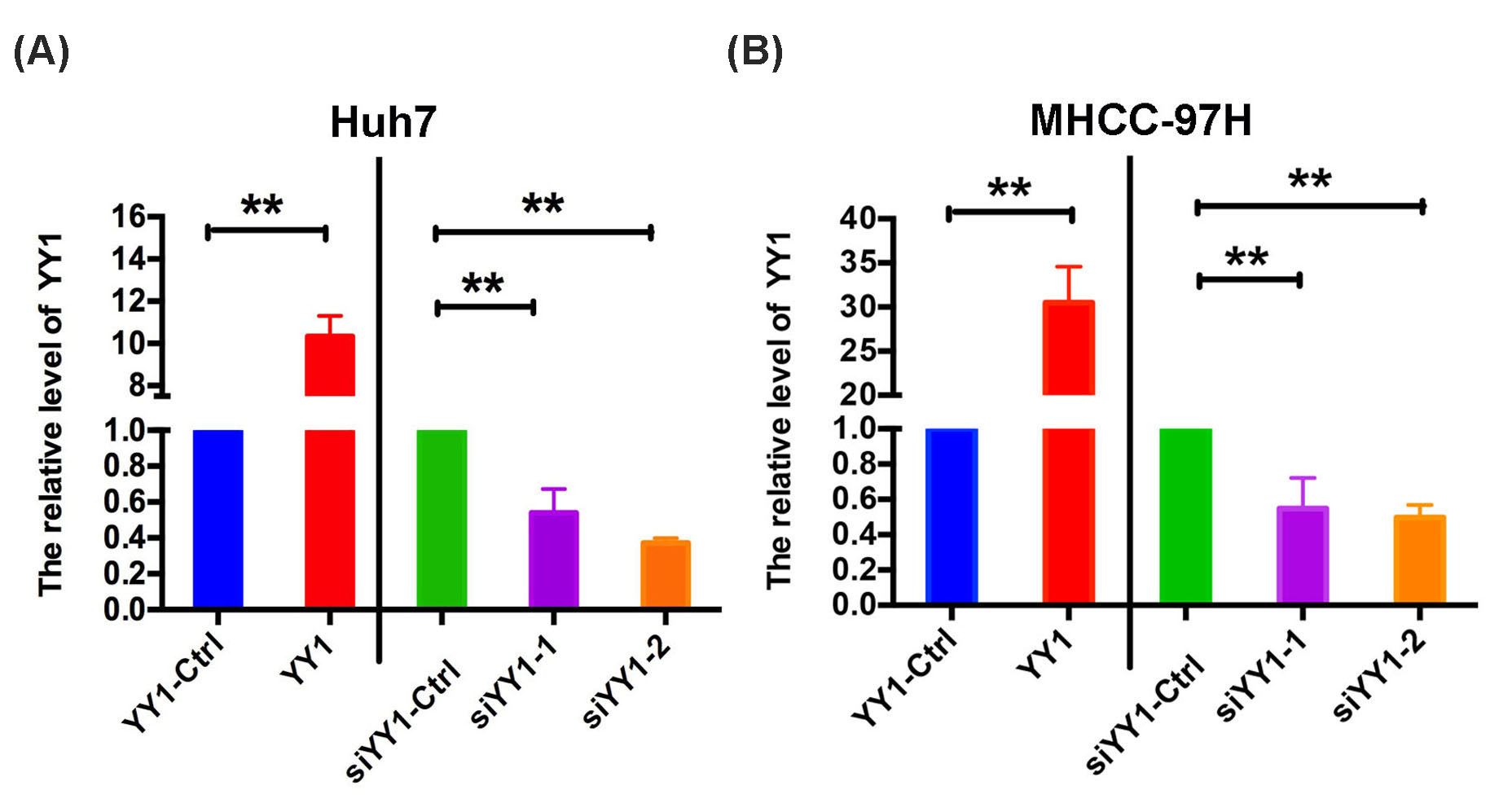

Supplement: Supplementary file 1 — Fig S1 [file CPR-53-e12835-s001.jpg]

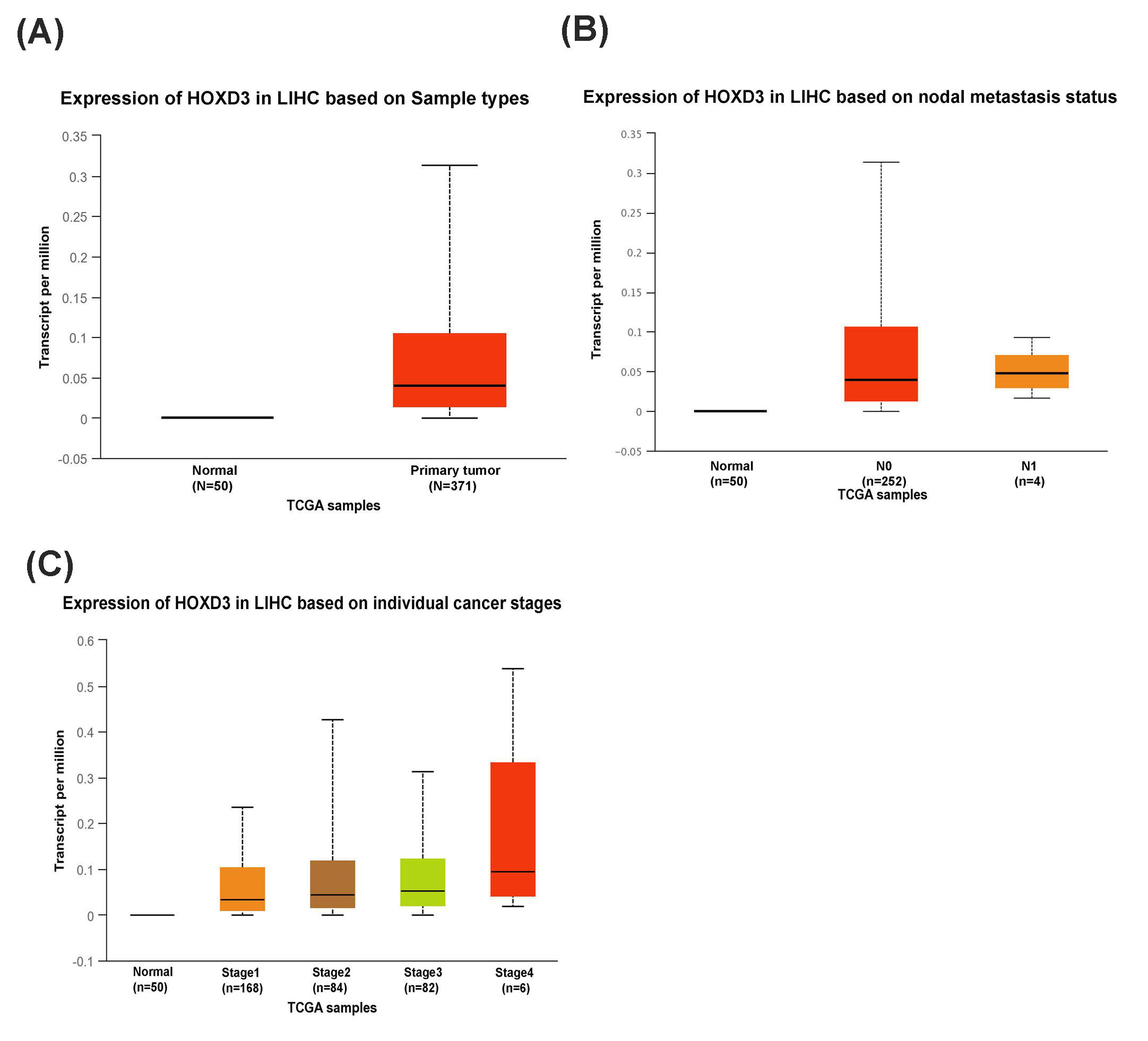

Supplement: Supplementary file 2 — Fig S2 [file CPR-53-e12835-s002.jpg]

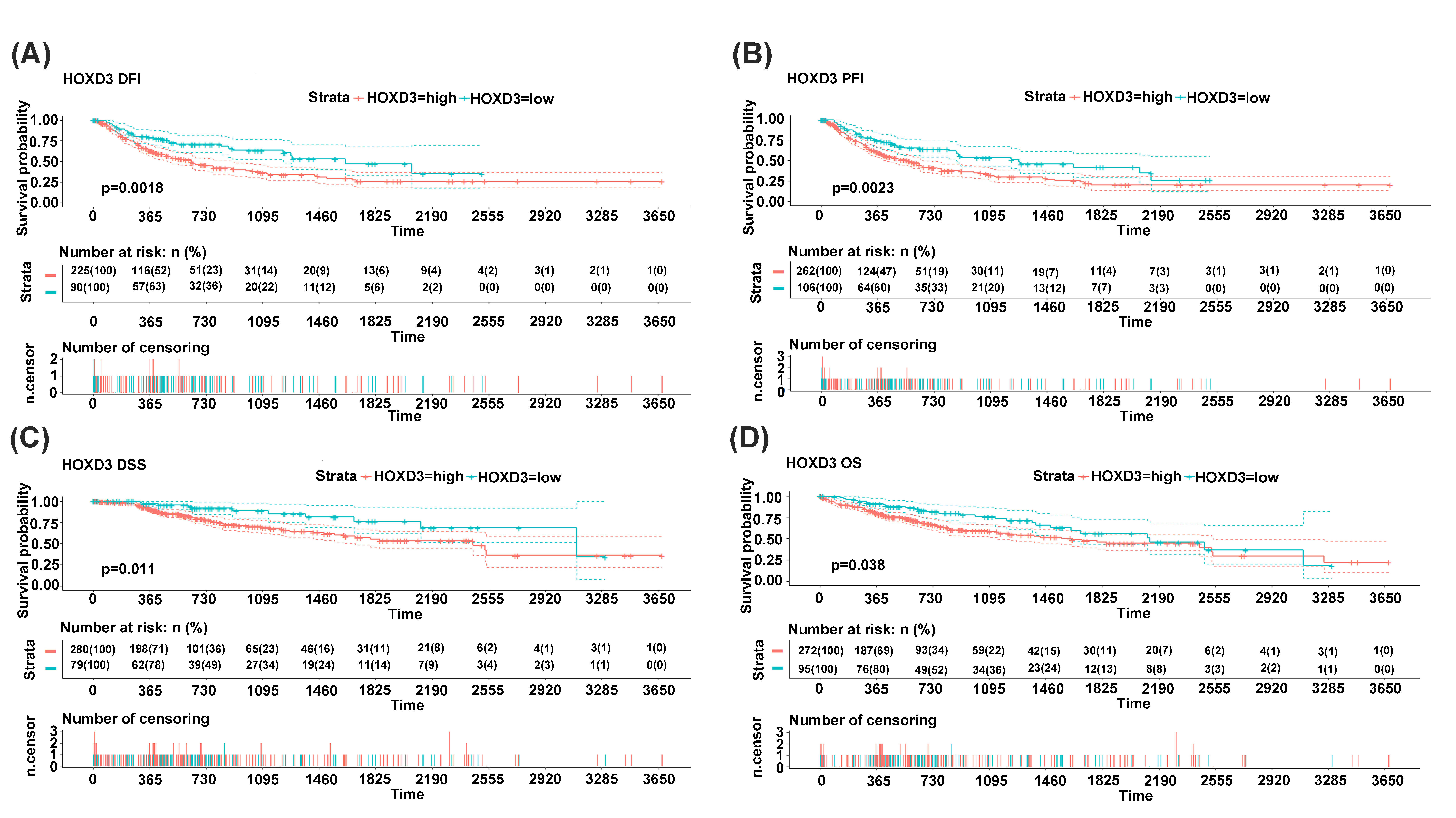

Supplement: Supplementary file 3 — Fig S3 [file CPR-53-e12835-s003.jpg]

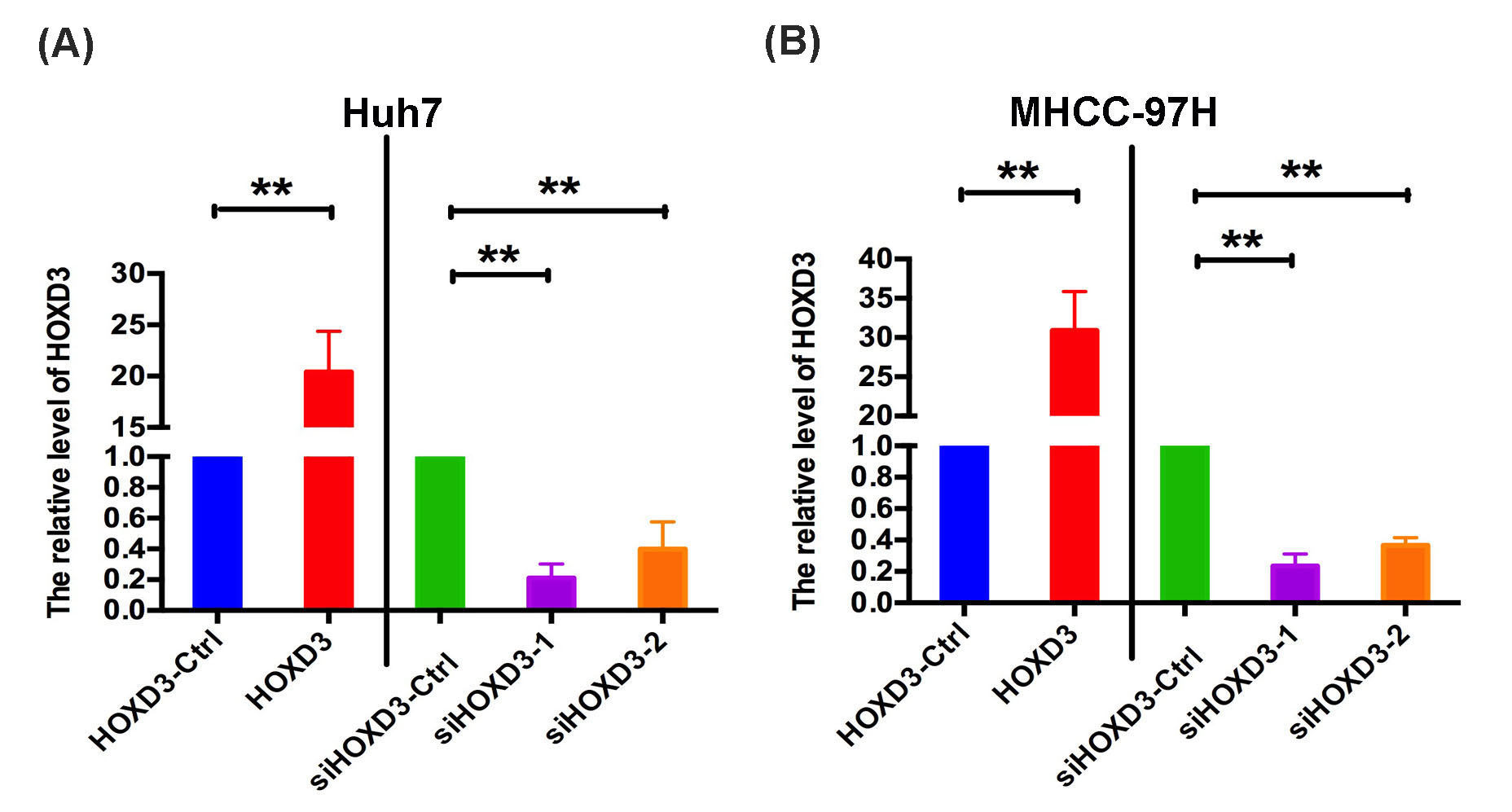

Supplement: Supplementary file 4 — Fig S4 [file CPR-53-e12835-s004.jpg]

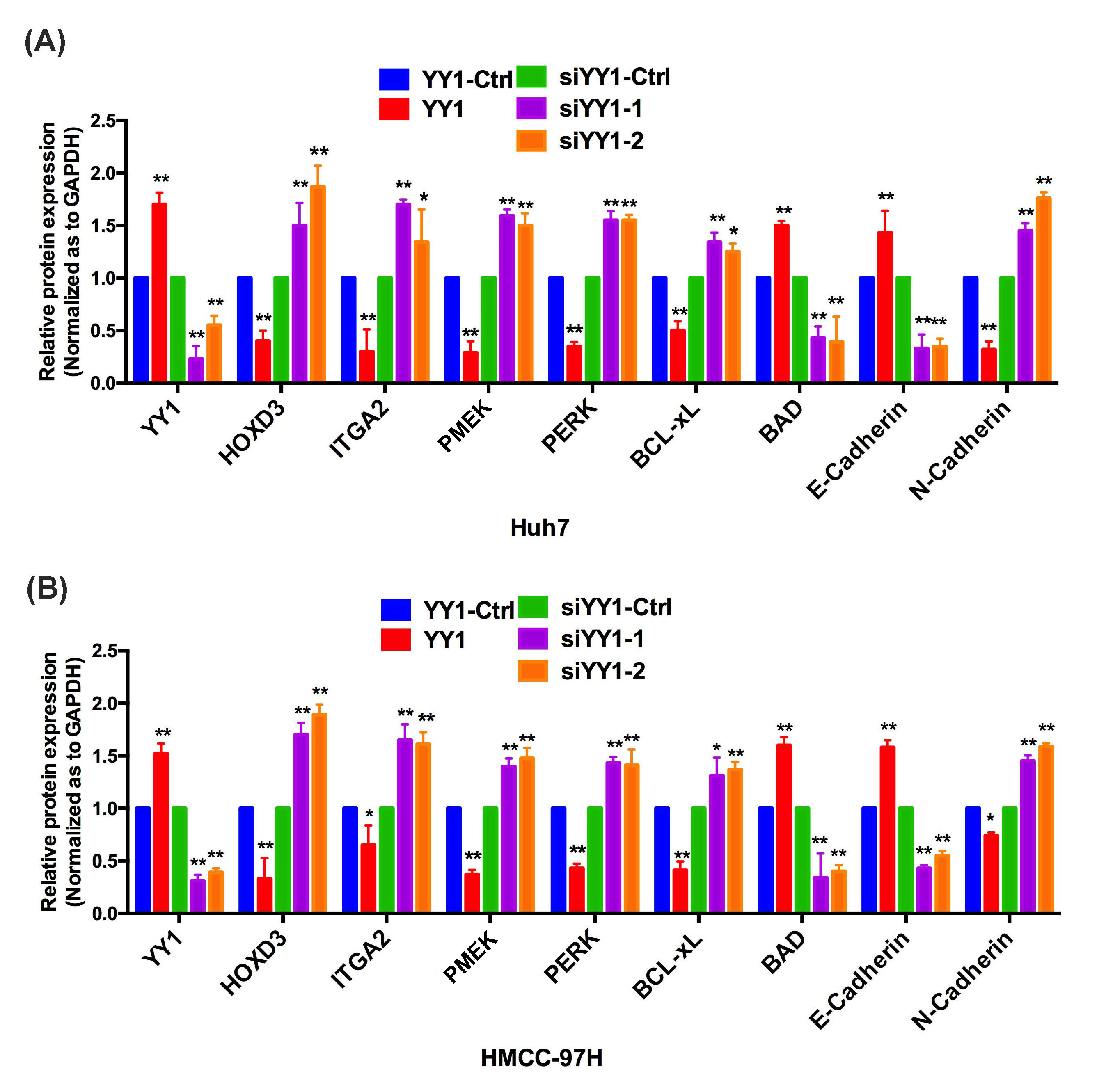

Supplement: Supplementary file 5 — Fig S5 [file CPR-53-e12835-s005.jpg]

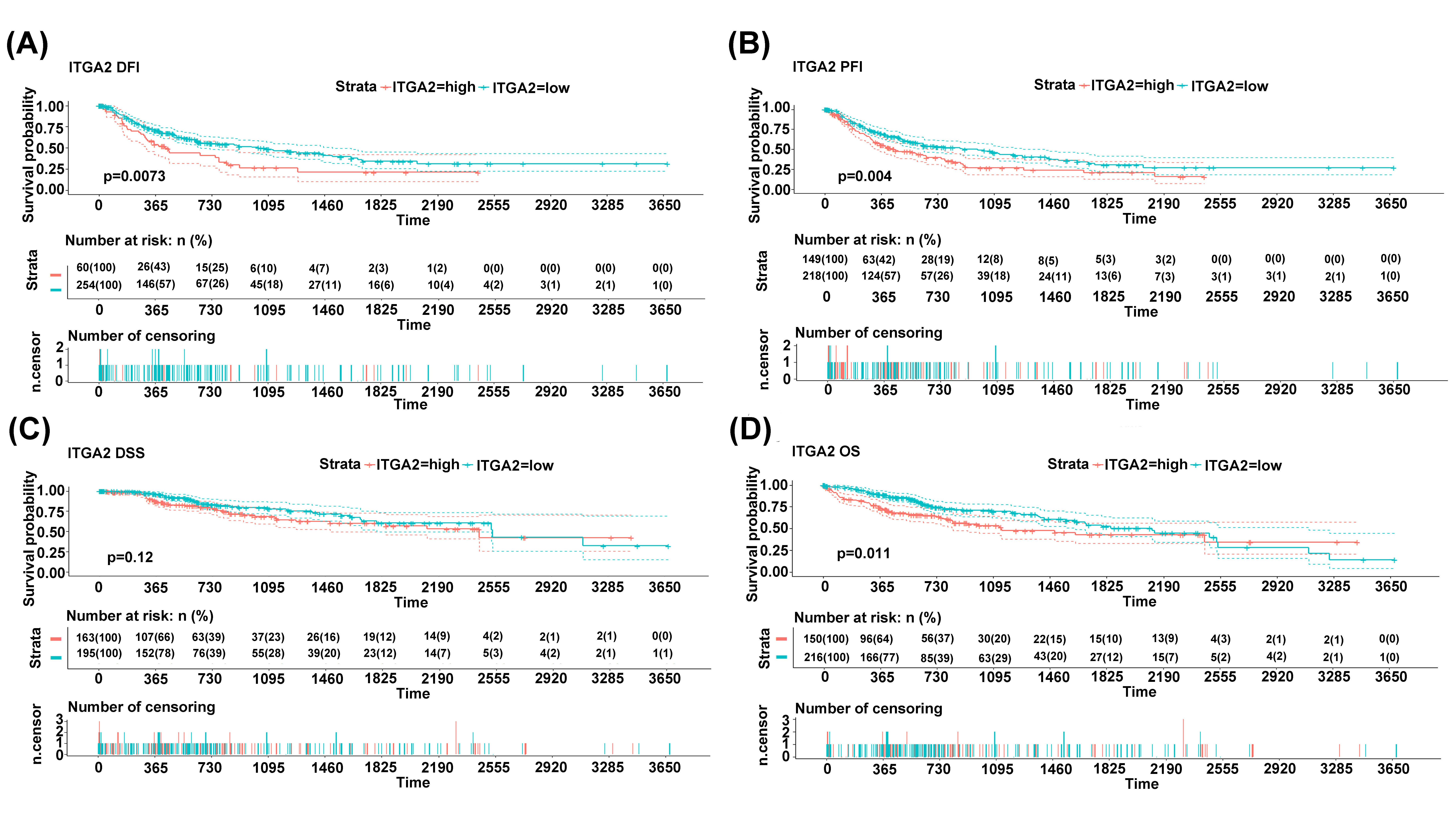

Supplement: Supplementary file 6 — Fig S6 [file CPR-53-e12835-s006.jpg]

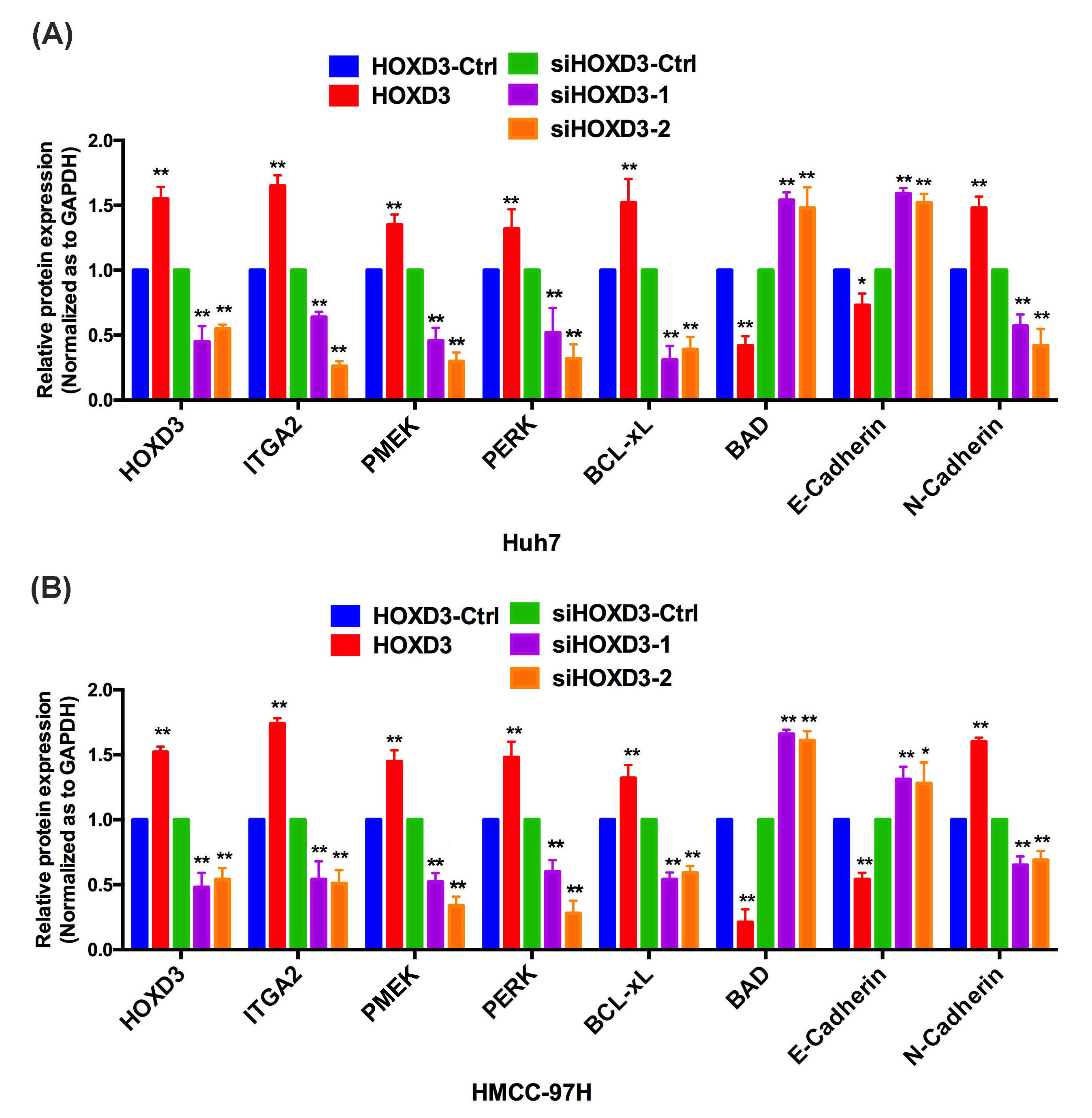

Supplement: Supplementary file 7 — Fig S7 [file CPR-53-e12835-s007.jpg]
