# Supplementary material for: HOXD3 was negatively regulated by YY1 recruiting HDAC1 to suppress progression of hepatocellular carcinoma cells via ITGA2 pathway
Source: Cell Prolif. 2020 Jun 17;53(8):e12835. doi: 10.1111/cpr.12835 (PMC7445403; doi:10.1111/cpr.12835)
Supplement: Supplementary file 8 — Table S1 [file CPR-53-e12835-s008.doc]

**Table S1.**

Primers and oligonucleotides used in this work.

| Name | Sequence(5’-3’) |
| --- | --- |
| siRNA-ctrl-S | 5′-UUCUCCGAACGUGUCACGUTT-3′ |
| siRNA-ctrl-A | 5′-ACGUGACACGUUCGGAGAATT-3′ |
| siHOXD3-1S | 5′-GAGUCUCGACAGAACUCCATT-3′ |
| siHOXD3-1A | 5′-UGGAGUUCUGUCGAGACUCTT-3′ |
| siHOXD3-2S | 5′-CAGUGGUAGUCAAUGUUAUUU-3′ |
| siHOXD3-2A | 5′-AUAACAUUGACUACCACUGUA-3′ |
| siYY1-1S | 5′-CGAUGGUUGUAAUAAGAAGUU-3′ |
| siYY1-1A | 5′-CUUCUUAUUACAACCAUCGAA-3′ |
| siYY1-2S | 5′-GAUGAUGCUCCAAGAACAAUA-3′ |
| siYY1-2A | 5′-UUGUUCUUGGAGCAUCAUCUU-3′ |
| CHIP-PCR HOXD3-S-1 | 5′-GAGGGAAGAACCACGAAGGG-3′ |
| CHIP-PCR HOXD3-A-1 | 5′-GCCAATGACGACCGCAAATA-3′ |
| CHIP-PCR HOXD3-S-2 | 5′-GGCTCTCCATTTGCAATGACC-3′ |
| CHIP-PCR HOXD3-A-2 | 5′-TCAACTTGGGGAATGAGGGC-3′ |
| CHIP-PCR HOXD3-S-3 | 5′- GGGGTTTGGCATCTGTTATTAA-3′ |
| CHIP-PCR HOXD3-A-3 | 5′-GCAGAGAGCTCTTCGATGACT-3′ |
| CHIP-PCR ITGA2-S-1 | 5′-TCTCTTTCCAGATGTGAGACTGA-3′ |
| CHIP-PCR ITGA2-A-1 | 5′-GCTGTGTGTGTGTGTGTGTG-3′ |
| CHIP-PCR ITGA2-S-2 | 5′-TCCAGAATTATCTCCTGTTTTTCT-3′ |
| CHIP-PCR ITGA2-A-2 | 5′-GGGAGAAGAGTGGCATTGCT-3′ |
| CHIP-PCR ITGA2-S-3 | 5′-CAGAGGTGAGTTGGAGCCAA-3′ |
| CHIP-PCR ITGA2-A-3 | 5′-CCCTTTTGATCTTTCACCATGGT-3′ |
| YY1-S | 5′-TGGAGAGAACTCACCTCCTGA-3′ |
| YY1-A | 5′-TCTTTAATTTTTCTTGGCTTCATTC-3′ |
| ITGA2-S | 5′- TCGTGCACAGTTTTGAAGATG -3′ |
| ITGA2-A | 5′- TGGAACACTTCCTGTTGTTACC -3′ |
| HOXD3-F | 5′-TCAAGAAAACACACACATACATAATTG-3′ |
| HOXD3-R | 5′-TGCTGAATCCTGAGAGAGCTG -3′ |
| GAPDH-F | 5′-GGAGCGAGATCCCTCCAAAAT-3′ |
| GAPDH-R | 5′- GGCTGTTGTCATACTTCTCATGG-3′ |
