# Supplementary material for: HOXD3 was negatively regulated by YY1 recruiting HDAC1 to suppress progression of hepatocellular carcinoma cells via ITGA2 pathway
Source: Cell Prolif. 2020 Jun 17;53(8):e12835. doi: 10.1111/cpr.12835 (PMC7445403; doi:10.1111/cpr.12835)
Supplement: Supplementary file 9 — Table S2 [file CPR-53-e12835-s009.docx]

**Table S2:**

**DATA and R language description**

1. **TCGA data download website and citation**

**website** :TCGA data download from UCSC XENA (https://xena.ucsc.edu)

**Cite:**

The UCSC Xena platform for public and private cancer genomics data visualization and interpretation

Mary Goldman, Brian Craft, Mim Hastie, Kristupas Repečka, Fran McDade, Akhil Kamath, Ayan Banerjee, Yunhai Luo, Dave Rogers, View ORCID ProfileAngela N. Brooks, Jingchun Zhu, David Haussler doi: <https://doi.org/10.1101/326470>

1. **The dataset of mRNA expression**

**dataset:** gene expression RNAseq - IlluminaHiSeq

**hub:** https://tcga.xenahubs.net

TCGA liver hepatocellular carcinoma (LIHC) gene expression by RNAseq (polyA+ IlluminaHiSeq)

**dataset ID:** TCGA.LIHC.sampleMap/HiSeqV2

download: https://tcga.xenahubs.net/download/TCGA.LIHC.sampleMap/HiSeqV2.gz; Full metadata

**samples:** 423

**version:** 2017-10-13

**type of data:** gene expression RNAseq

**unit:** log2(norm_count+1)

**platform:** IlluminaHiSeq_RNASeqV2

**ID/Gene Mapping:** <https://tcga.xenahubs.net/download/probeMap/>

hugo_gencode_good_hg19_V24lift37_probemap; Full metadata

**author:** University of North Carolina TCGA genome characterization center

**raw data:** <https://tcga-data.nci.nih.gov/tcgafiles/ftp_auth/distro_ftpusers/anonymous/>

tumor/lihc/cgcc/unc.edu/illuminahiseq_rnaseqv2/rnaseqv2/

**wrangling:** Level_3 data (file names: *.rsem.genes.normalized_results) are downloaded from TCGA DCC, log2(x+1) transformed, and processed at UCSC into Xena repository

1. **The dataset of clinical stage：**

**dataset:** phenotype - Phenotypes

**hub:** https://tcga.xenahubs.netcohort

**dataset ID:** TCGA.LIHC.sampleMap/LIHC_clinicalMatrix

download: https://tcga.xenahubs.net/download/TCGA.LIHC.sampleMap/LIHC_clinicalMatrix; Full metadata

**samples:** 438

**version:** 2019-12-06

**type of data:** phenotype

**raw data:** <https://tcga> data.nci.nih.gov/tcgafiles/ftp_auth/distro_ftpusers/anonymous/tumor/lihc/bcr/

**Notes of different clinical stage in manuscript**

neoplasm_histologic_grade: G1 G2 G3 G4

pathologic_stage: Stage I Stage II Stage III Stage IV

pathologic_T: T1 T2 T3 T4

pathologic_M: M0 M1

pathologic_N: N0 N1

1. **The survival dataset：**

**dataset:** phenotype - Curated survival data

**hub:** https://tcga.xenahubs.net

Curated survival data from the Pan-cancer Atlas paper titled "An Integrated TCGA Pan-Cancer Clinical Data Resource (TCGA-CDR) to drive high quality survival outcome analytics". The paper highlights four types of carefully curated survival endpoints, and recommends the use of the endpoints of OS(overall survial), PFI( progression-free interval), DFI(disease-free interval), and DSS(disease-specific survival) for each TCGA cancer type.

**dataset ID:** survival/LIHC_survival.txt

download: https://tcga.xenahubs.net/download/survival/LIHC_survival.txt.gz; Full metadata

**samples:** 438

**version:** 2018-09-13

**type of data:** phenotype

**raw data:** http://www.cell.com/cell/fulltext/S0092-8674(18)30229-0

1. **the version and citation of ggstatsplot**

**Version:** 0.2.0

**Published:** 2020-02-03

**Cite:**

Patil I (2018). ggstatsplot: 'ggplot2' Based Plots with Statistical Details. doi: 10.5281/zenodo.2074621, <https://CRAN.R-project.org/package=ggstatsplot>.

**Data comparison in different group**

ggbetweenstats drawing

a. two groups：t-test

b. more than two groups：anova

1. **the version and citation of survival**

**Version:** 3.1-8

**Published:** 2019-12-03

**Cite:**

Therneau T (2015). A Package for Survival Analysis in S. version 2.38, https://CRAN.R-project.org/package=survival.

Survfit: Kaplane Meier

Surv cutpoint: identify a best cutoff point to divide the cancer patients as high/low gene expression groups

1. **the version and citation of survminer**

**Version:** 0.4.6

**Published:** 2019-09-03

**Cite:**

Alboukadel Kassambara, Marcin Kosinski and Przemyslaw Biecek (2019). survminer: Drawing Survival Curves using 'ggplot2'. R package version 0.4.6. https://CRAN.R-project.org/package=survminer

1. **the version and citation of ggrisk**

**Version:** 1.0

**Published:** 2020-02-09

**Cite:**

Jing Zhang and Zhi Jin (2020). ggrisk: Risk Score Plot for Cox Regression. R package version 1.0. https://CRAN.R-project.org/package=ggrisk
