# Supplementary material for: HOXD3 was negatively regulated by YY1 recruiting HDAC1 to suppress progression of hepatocellular carcinoma cells via ITGA2 pathway
Source: Cell Prolif. 2020 Jun 17;53(8):e12835. doi: 10.1111/cpr.12835 (PMC7445403; doi:10.1111/cpr.12835)
Supplement: Supplementary file 10 — Supplementary Material [file CPR-53-e12835-s010.docx]

**Figure S1** YY1 expressed in Huh7 and HMCC-97H cells. A, qRT-PCR analysis of YY1 in Huh7 transfected with YY1 overexpression construct and two RNA interference (small interfering RNA (siRNA)). B, qRT-PCR analysis of YY1 in HMCC-97H transfected with YY1 overexpression construct, si-YY1-1, si-YY1-2 and their respectively control (*: p < 0.05, **: p < 0.01).

**Figure S2** HOXD3 higher expressed in HCC tissues and induced metastasis of HCCs. A, TCGA database showed that HOXD3 expression in HCC tissues and their normal tissues. B, TCGA database showed that HOXD3 expression in HCC tissues and their normal tissues based on nodal metastasis status. C, TCGA database showed that HOXD3 expression in HCC tissues and their normal tissues based on individual cancer stages.

**Figure S3** The expression of HOXD3 induced the lower survival rate. A-D, the expression of HOXD3 was related with the liver cancer DFI (disease-free interval event), PFI (progression-free interval event), DSS (disease-specific survival event) and OS(overall survival).

**Figure S4** HOXD3 expressed in Huh7 and HMCC-97H cells. A, qRT-PCR analysis of HOXD3 in Huh7 transfected with HOXD3 overexpression vector and two RNA interference. B, qRT-PCR analysis of HOXD3 in HMCC-97H transfected with HOXD3, si-HOXD3-1, si-HOXD3-2 and their respectively control (*: p < 0.05, **: p < 0.01).

**Figure S5** Proteins expressed in Huh7 and HMCC-97H cells treated with YY1 and siYY1. A, Western blotting analysis of YY1, ITGA2, HOXD3, p-MEK, p-ERK, BCL-xL, BAD, E-Cadherin and N- Cadherin expression in Huh7 cells transfected with YY1-Ctrl, YY1, siYY1-Ctrl, siYY1-1 and siYY1-2. GAPDH was detected as an internal control. B, Western blotting analysis of YY1, ITGA2, HOXD3, p-MEK, p-ERK, BCL-xL, BAD, E-Cadherin and N- Cadherin expression in HMCC-97H cells transfected with YY1-Ctrl, YY1, siYY1-Ctrl, siYY1-1 and siYY1-2. GAPDH was detected as an internal control (*: p < 0.05, **: p < 0.01).

**Figure S6** The expression of ITGA2 induced the lower survival rate. A-D, the expression of HOXD3 was related with the liver cancer DFI (disease-free interval event), PFI (progression-free interval event), DSS (disease-specific survival event) and OS(overall survival).

**Figure S7** Proteins expressed in Huh7 and HMCC-97H cells treated with HOXD3 and siHOXD3. A, Western blotting analysis of HOXD3, ITGA2, p-MEK, p-ERK, BCL-xL, BAD, E-Cadherin and N- Cadherin expression in Huh7 cells transfected with HOXD3-Ctrl, HOXD3, siHOXD3-Ctrl, siHOXD3-1 and siHOXD3-2. GAPDH was detected as an internal control. B, Western blotting analysis of HOXD3, ITGA2, p-MEK, p-ERK, BCL-xL, BAD, E-Cadherin and N-Cadherin expression in HMCC-97H cells transfected with HOXD3-Ctrl, HOXD3, siHOXD3-Ctrl, siHOXD3-1 and siHOXD3-2. GAPDH was detected as an internal control (*: p < 0.05, **: p < 0.01).
